# Qualitative analysis of 7- and 8-hydroxyzolpidem and discovery of novel zolpidem metabolites in postmortem urine using liquid chromatography–tandem mass spectrometry

**DOI:** 10.1007/s11419-021-00611-9

**Published:** 2022-01-04

**Authors:** Koji Yamaguchi, Hajime Miyaguchi, Youkichi Ohno, Yoshimasa Kanawaku

**Affiliations:** 1 Department of Legal Medicine, Nippon Medical School, 1715 Kamagari, Inzai, Chiba 270-1694 Japan; 2 National Research Institute of Police Science, 6-3-1 Kashiwanoha, Kashiwa, Chiba 277-0882 Japan

**Keywords:** Zolpidem, Structural elucidation, LC-QqQMS, LC-QqTOFMS, Dihydrodiol, Cysteine adducts

## Abstract

**Purpose:**

Zolpidem (ZOL) is a hypnotic sometimes used in drug-facilitated crimes. Understanding ZOL metabolism is important for proving ZOL intake. In this study, we synthesized standards of hydroxyzolpidems with a hydroxy group attached to the pyridine ring and analyzed them to prove their presence in postmortem urine. We also searched for novel ZOL metabolites in the urine sample using liquid chromatography–triple quadrupole mass spectrometry (LC-QqQMS) and liquid chromatography–quadrupole time-of-flight mass spectrometry (LC-QqTOFMS).

**Methods:**

7- and 8-Hydroxyzolpidem (7OHZ and 8OHZ, respectively) were synthesized and analyzed using LC-QqQMS. Retention times were compared between the synthetic standards and extracts of postmortem urine. To search for novel ZOL metabolites, first, the urine extract was analyzed with data-dependent acquisition, and the peaks showing the characteristic fragmentation pattern of ZOL were selected. Second, product ion spectra of these peaks at various collision energies were acquired and fragments that could be used for multiple reaction monitoring (MRM) were chosen. Finally, MRM parameters were optimized using the urine extract. These peaks were also analyzed using LC-QqTOFMS.

**Results:**

The presence of 7OHZ and 8OHZ in urine was confirmed. The highest peak among hydroxyzolpidems was assigned to 7OHZ. The novel metabolites found were zolpidem dihydrodiol and its glucuronides, cysteine adducts of ZOL and dihydro(hydroxy)zolpidem, and glucuronides of hydroxyzolpidems.

**Conclusions:**

The presence of novel metabolites revealed new metabolic pathways, which involve formation of an epoxide on the pyridine ring as an intermediate.

**Supplementary Information:**

The online version contains supplementary material available at 10.1007/s11419-021-00611-9.

## Introduction

Hypnotics are used to treat insomnia, but they are sometimes involved in drug-facilitated crimes including sexual assaults, homicides, and robberies. In such cases, forensic toxicologists are asked to analyze the hypnotics and/or their metabolites in body fluids, such as blood, urine, and saliva, obtained from a victim. Understanding the metabolism of hypnotics is necessary for demonstrating misuse. In addition, information about drug intake can be obtained; for example, the time interval after drug intake can be estimated from concentration ratios of hypnotic metabolites. Forsman et al. investigated urinary excretion patterns of flunitrazepam and its metabolites, and concluded that the ratio of 7-aminodesmethylflunitrazepam to 7-aminoflunitrazepam may be used to estimate the time of intake [1]. Tsujikawa et al. examined the urinary excretion patterns of triazolam metabolites and found that the ratio of *α*-hydroxytriazolam to 4-hydroxytriazolam were decreased over time [2]. Feng et al. analyzed zolpidem (ZOL) and its major metabolite (M1) in oral fluid and found that ZOL/M1 ratio decreased after ZOL intake [3]. These studies demonstrated the usefulness of analysis of hypnotic metabolites. However, prior to studies about concentrations of hypnotic metabolites in human body fluids, identification of hypnotic metabolites and preparation of their analytical standards are required.

In the past few decades, liquid chromatography–tandem mass spectrometry (LC–MS/MS) utilizing the triple quadrupole (QqQ) and quadrupole-time-of-flight (QqTOF) analyzers has become widely used in forensic toxicological analysis because of its excellent selectivity and sensitivity [4, 5]. Now drug metabolites can be found that are produced from only a small fraction of an administered drug. By applying these technologies, novel metabolites not known at the time of launch can be discovered even for well-known hypnotics, such as Z-drugs (ZOL, and zopiclone) and benzodiazepines. Novel metabolites can show different characteristics in the time courses of blood concentration, distribution, and excretion, and these findings enable further understanding of drug metabolism. For example, we previously found novel quazepam metabolites in human bile and revealed that some of them accumulate in bile at extremely high concentrations [6].

**Fig. 1:**
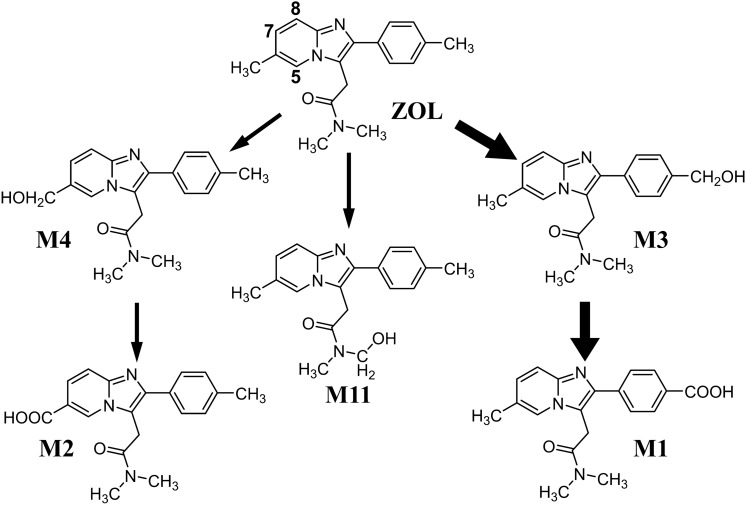
Metabolic pathway of ZOL

ZOL is a short-acting hypnotic and is the most widely used prescription drug for sleep disorders in the Unites States [7]. Accordingly, the analysis of ZOL and its metabolites in biological samples is a major concern in forensic toxicology. The metabolism of ZOL, reported from a study in the drug development process, is illustrated in Fig. 1 [8]. In our previous work, we developed a synthetic method for ZOL metabolites and successfully synthesized M1–M4 [9]. We also analyzed ZOL metabolites in human blood and urine using LC-QqQMS, and revealed the presence of an unknown metabolite, which is a hydroxyzolpidem with a hydroxy group on the pyridine ring. However, the exact location of the hydroxy group could not be elucidated by LC-QqQMS alone. There are three possible locations, namely, the 5-, 7-, and 8-positions as depicted in Fig. 1. In this study, we elucidated the exact location of the hydroxy group by synthesizing standards of hydroxyzolpidems. We also searched for novel ZOL metabolites in postmortem urine using LC-QqQMS and LC-QqTOFMS.

## Materials and methods

### Reagents

*N*,*N*-Dimethylpropiolamide (DMPA, IUPAC name: *N*,*N*-dimethylprop-2-ynamide) was synthesized as described previously [9]. The precursor materials for the synthesis of 7-methoxyzolpidem (7OMeZ) and 8-benzyloxyzolpidem (8OBzZ), 2-amino-4-methoxy-5-methylpyridine (**1**) and 2-amino-3-benzyloxy-5-methylpyridine (**2**), respectively, were synthesized in this study. The details of synthesis are presented in the supplementary information. Pyrrolidine and 1 mol/L methylene chloride solution of boron tribromide (BBr_3_) were purchased from Tokyo Chemical Industry Co. Ltd. (Tokyo, Japan). Copper(I) chloride (CuCl), copper(II) triflate (Cu(OTf)_2_), 0.1 mol/L phosphate buffer (pH 6), molecular sieves 4A, and NH_2_ silica gel (Wakogel, 50NH_2_) were purchased from Fujifilm Wako Pure Chemical Co. (Osaka, Japan). *β*-Glucuronidase (from *Escherichia coli*., type IX-A) was purchased from Sigma-Aldrich (St. Louis, MO). Other chemicals were purchased from Kanto Chemical Co., Inc. (Tokyo, Japan).

### Nuclear magnetic resonance spectroscopy

^1^H and ^13^C nuclear magnetic resonance (NMR) spectra were recorded using an Avance III HD 400 (Brucker Co., Billerica, MA) or ECA-600 (JEOL Ltd., Tokyo, Japan) spectrometer. Chemical shifts were referenced to the tetramethylsilane (TMS) signal at *δ* = 0 ppm in chloroform-*d* (CDCl_3_) or to the residual solvent signals at *δ* = 2.50 and 39.52 ppm in dimethylsulfoxide-*d*_6_ (DMSO-*d*_6_) for ^1^H and ^13^C NMR spectra, respectively. All NMR spectra of the compounds synthesized in this study are presented in the supplementary information.

### High-resolution mass spectrometry for synthesized compounds

High-resolution mass spectrometry (HR-MS) measurements for synthesized compounds were performed using a Q Exactive mass spectrometer (Thermo Fisher Scientific, Waltham, MA) with an electrospray ionization (ESI) probe. Full scan mass spectra were recorded in positive mode. The conditions for ESI were as follows: sheath gas flow rate, 5 units; capillary temperature, 320 °C; spray voltage, 3.5 kV; probe heater temperature, 350 °C; S-lens RF level, 50 units. Products were dissolved in a 1:1 (*v*/*v*) mixture of water–acetonitrile containing 0.1% formic acid at a concentration of 1 μg/mL and infused into the spectrometer at a flow rate of 10 μL/min.

### General procedure of the modified three-component coupling reaction

A three-component coupling reaction was performed using a previously reported procedure with modification [9]. Molecular sieves 4A (2 g), 4-methylbenzaldehyde (2 mmol), 2-aminopyridine (**1** or **2**, 2 mmol), chloroform (4 mL), and pyrrolidine (0.2 mmol) were placed in a 30 mL screw vial. The mixture was sealed and heated at 100 °C for 3 h. After cooling to room temperature, CuCl (0.4 mmol), Cu(OTf)_2_ (0.4 mmol), and DMPA (3.6 mmol) were added. The vial was sealed again and heated at 80 °C for 3 h. After removing the molecular sieves by filtration, ammonia (28%, 0.4 mL) was added to the solution and the mixture was stirred at room temperature for 10 min. The mixture was passed through an NH_2_ silica gel column (3 cm [i.d.] × 1 cm [height]), and the column was washed with chloroform (30 mL). The solvent was removed by evaporation and the product was purified by silica gel column chromatography (eluent: chloroform–ethyl acetate 1:1 for 7OMeZ and 3:1 for 8OBzZ). Recrystallization from ethyl acetate–diisopropyl ether for 7OMeZ and chloroform–ethyl acetate for 8OBzZ gave the desired products as white crystals.

7OMeZ: Yield 44%. ^1^H NMR (400 MHz, CDCl_3_) *δ* 2.21 (s, 3H), 2.40 (s, 3H), 2.86 (s, 3H), 2.94 (s, 3H), 3.88 (s, 3H), 4.07 (s, 2H), 6.90 (s, 1H), 7.25 (m, 2H), 7.54 (m, 2H), 7.96 (s, 1H). ^13^C NMR (151 MHz, CDCl_3_) *δ* 14.00, 21.28, 30.40, 35.86, 37.52, 55.47, 93.61, 112.08, 116.14, 123.11, 128.27, 129.32, 132.14, 137.17, 142.80, 146.15, 157.47, 168.65. Elemental analysis calcd for C_20_H_23_N_3_O_2_: C 71.19%, H 6.87%, N 12.45%; found: C 70.72%, H 6.95%, N 12.02%. HR-MS: calcd for C_20_H_24_N_3_O_2_ ([M + H]^+^) 338.1863; found 338.1855.

8OBzZ: Yield 42%. ^1^H NMR (400 MHz, CDCl_3_) *δ* 2.26 (s, 3H), 2.39 (s, 3H), 2.87 (s, 3H). 2.94 (s, 3H), 4.07 (s, 2H), 5.35 (s, 2H), 6.35 (s, 1H), 7.24 (m, 2H), 7.2–7.5 (m, 5H), 7.56 (m, 2H), 7.66 (s, 1H). ^13^C NMR (151 MHz, CDCl_3_) *δ* 18.96, 21.29, 30.60, 35.86, 37.49, 70.58, 106.29, 114.65, 115.45, 121.66, 127.26, 127.88, 128.55, 128.76, 129.19, 131.92, 136.63, 137.28, 138.70, 143.26, 147.10, 168.40. Elemental analysis calcd for C_26_H_27_N_3_O_2_: C 75.52%, H 6.58%, N 10.16%; found: C 75.31%, H 6.66%, N 10.06%. HR-MS: calcd for C_26_H_28_N_3_O_2_ ([M + H]^+^) 414.2176; found 414.2168.

### Synthesis of 7OHZ hydrobromide

7OMeZ (337 mg, 1.00 mmol) was dissolved in chloroform (4 mL) and then 1 mol/L BBr_3_ solution (4 mL) was added. The mixture was heated at 60 °C and stirred for 15 h. Acetonitrile containing 20% (*v*/*v*) of water (5 mL) was added and the mixture was stirred for 10 min at room temperature. The reaction mixture was evaporated to dryness. The product was purified by column chromatography (silica gel, eluent: chloroform‒ethanol‒formic acid 90:10:1). The fractions containing the desired product were pooled and evaporated. The desired product was recrystallized from isopropanol‒water as white crystals. Yield 0.264 g (0.652 mmol, 65%). ^1^H NMR (400 MHz, DMSO-*d*_6_) *δ* 2.23 (s, 3H), 2.39 (s, 3H), 2.90 (s, 3H), 3.13 (s, 3H), 4.20 (s, 2H), 7.05 (s, 1H), 7.42 (m, 2H,), 7.45 (m, 2H), 8.52 (s, 1H,), 12.10 (brs, 1H), 13.70 (brs, 1H). ^13^C NMR (151 MHz, DMSO-*d*_6_) *δ* 13.09, 20.86, 28.07, 35.32, 36.95, 91.42, 116.35, 119.60, 124.19, 126.48, 127.75, 129.96, 130.87, 139.59, 140.43, 161.52, 166.96. Elemental analysis calcd for C_19_H_22_BrN_3_O_2_: C 56.44%, H 5.48%, N 10.39%; found: C 56.27%, H 5.32%, N 10.20%. HR-MS: calcd for C_19_H_22_N_3_O_2_ ([M + H]^+^) 324.1707; found 324.1700.

### Synthesis of 8OHZ tosylate

8OBzZ (209 mg, 0.505 mmol) was dissolved in chloroform (2 mL), mixed with BBr_3_ solution (2 mL, 2 mmol), heated at 60 °C, and stirred for 15 h. The mixture was hydrolyzed as described in the synthesis of 7OHZ. The mixture was neutralized with saturated aqueous sodium bicarbonate and evaporated to dryness. The desired product was purified by column chromatography (NH_2_ silica gel, eluent: chloroform‒ethanol 5:1), and fractions containing the desired product were pooled, mixed with *p*-toluene sulfonic acid monohydrate (122 mg, 0.641 mmol), and evaporated to dryness. Recrystallization from acetonitrile–ethyl acetate afforded white crystals. Yield 100 mg (0.203 mmol, 40%). ^1^H NMR (400 MHz, DMSO-*d*_6_) *δ* 2.28 (s, 3H), 2.38 (s, 3H), 2.40 (s, 3H), 2.90 (s, 3H), 3.12 (s, 3H), 4.21 (s, 2H), 7.06 (s, 1H), 7.08–7.12 (m, 2H), 7.39–7.42 (m, 2H) 7.46–7.50 (m, 4H), 8.09 (s, 1H,), 12.01 (brs, 1H), 14.78 (brs, 1H). ^13^C NMR (100 MHz, DMSO-*d*_6_) *δ* 17.88, 20.75, 20.91, 28.42, 35.33, 36.96, 115.14, 115.86, 118.81, 123.72, 125.47, 127.42, 128.03, 128.65, 129.72, 132.05, 133.20, 137.64, 139.85, 142.22, 145.64, 166.80. Elemental analysis calcd for C_26_H_29_N_3_O_5_S: C 63.01%, H 5.90%, N 8.48%; found: C 62.95%, H 5.96%, N 8.37%. HR-MS: calcd for C_19_H_22_N_3_O_2_ ([M + H]^+^) 324.1707; found 324.1701.

### Urine sample

The deceased was a man in his eighties and a victim of fire. He had been prescribed Myslee^®^ 5 mg tablets containing 5 mg of ZOL tartrate per tablet about 2 weeks before his death. The deceased’s urine and heart blood were collected during a medico-legal autopsy. The cause of death was carbon monoxide poisoning; the carboxyhemoglobin saturation in the heart blood was more than 75% using Avoximeter 4000 oximeter (International Technidyne Co., Edison, NJ). The results of our routine drug screening analysis revealed that the ZOL concentration in the heart blood was at the therapeutic level (61 ng/mL).

### Sample pretreatment

The urine (400 μL) was mixed with 0.1% formic acid–acetonitrile (800 μL) and centrifuged (6800×*g*, 5 min). The supernatant was evaporated under a nitrogen stream at 40 °C. The residue was dissolved in methanol (100 μL), mixed with water (100 μL), and centrifuged (6800×*g*, 5 min). The supernatant was transferred to a vial and 10 μL aliquots were injected into the LC-QqQMS and LC-QqTOFMS systems.

### LC-QqQMS conditions

LC-QqQMS analysis was performed using a Nexera XR high-performance liquid chromatograph (Shimadzu Co., Kyoto, Japan) equipped with an LCMS-8040 mass spectrometer (Shimadzu Co.). Chromatographic separation was achieved on an InertSustain C18 column (length 250 mm, i.d. 2.1 mm, particle size 3 μm; GL Sciences Inc., Tokyo, Japan) at 40 °C. The mobile phase consisted of 10 mM aqueous ammonium formate (A) and methanol (B) at a flow rate of 0.3 mL/min. The gradient program was as follows: linear gradient from 10 to 40% B at 0‒3 min, from 40 to 50% B at 3‒18 min, from 50 to 70% B at 18‒25 min, from 70 to 98% B at 25‒26 min, isocratic elution at 98% B at 26‒30 min, re-equilibration with 10% B for 5 min. The total runtime was 35 min. Ionization was performed with ESI in positive mode. The conditions for ESI were as follows: nebulizer gas flow rate, 3 L/min; drying gas flow rate, 15 L/min; desolvation line temperature, 250 °C; heating block temperature, 400 °C; and source voltage, 5 kV. Product ion scan mode was applied to acquire product ion spectra of 7OHZ and 8OHZ with precursor ion *m*/*z* 324.30, collision energies (CEs) 15, 35, 55, and 75 V, and scan range *m*/*z* 10–340. Multiple reaction monitoring (MRM) mode was applied to analyze ZOL, M1, M2, and hydroxyzolpidems, and the parameters are listed in Table 1.

**Table 1:** Parameters of precursor ions, product ions, and CEs in MRM mode for ZOL, M1 and M2, and hydroxyzolpidems

Analytes	Precursor ions (*m*/*z*)	Product ions (*m*/*z*)	CE (V)
ZOL	308.10	235.10	36
		236.15	30
		263.15	27
M1 and M2	338.20	265.10	38
		293.10	28
		266.15	30
Hydroxyzolpidems	324.30	251.20	36
		279.15	25
		252.15	14

*CE* collision energy, *MRM* multiple reaction monitoring

To search for ZOL metabolites, first, data-dependent acquisition (DDA) mode was applied. The scan range of the trigger scan was *m*/*z* 100–800. Product ion spectra were acquired at a CE of 35 V over *m*/*z* 20–800. All product ion spectra acquired were checked and peaks that showed characteristic fragmentation patterns (loss of NMe_2_ and NMe_2_CO, − 45 and − 73 Da, respectively) were selected. Second, these peaks were analyzed by product ion scan mode with various CEs (15, 35, 55, and 75 V). Fragment ions with higher intensities were chosen, and finally, MRM parameters for those transitions were optimized by injecting the urine extract. Parameters for the new metabolites (A–E) are listed in Table 2.

**Table 2:** Precursor ions, product ions, and CEs in MRM mode, and scan ranges in product ion scan mode for peaks A–E

Peak	Precursor ions (*m*/*z*)	MRM mode		Product ion scan mode	Assignment
		Product ions (*m*/*z*)	CE (V)	Scan ranges (*m*/*z*)	
A	342.0	251.2	30	10‒360	ZDHD
		279.2	29		
		235.3	48		
B	427.0	340.1	24	10‒440	Z-Cys
		266.9	48		
		294.9	35		
C	445.1	324.1	18	10–460	Z-H_2_OCys
		251.2	41		
		339.9	28		
D	500.0	324.1	25	10‒540	Z-OGlu
		251.2	53		
		279.1	50		
E	518.0	251.1	43	10‒560	ZDHD-Glu
		324.1	24		
		342.1	27		

CEs for product ion scans were set to 15, 35, 55, and 75 V (see text)

*CE* collision energy, *MRM* multiple reaction monitoring

### LC-QqTOFMS conditions

LC-QqTOFMS analysis was performed using a Nexera X3 high-performance liquid chromatograph (Shimadzu Co.) equipped with an LCMS-9030 mass spectrometer (Shimadzu Co.). Chromatographic separation was carried out by applying the same conditions of those for LC-QqQMS analysis. Ionization was performed by ESI in positive mode under the following conditions: nebulizer gas flow rate, 3 L/min; heating gas flow rate, 10 L/min; drying gas flow rate, 15 L/min; interface temperature, 300 °C; desolvation line temperature, 250 °C; heating bloc temperature, 400 °C; and interface voltage, 4 kV. The DDA mode was applied. The scan range of the trigger scan was *m*/*z* 200–800. Product ion spectra were acquired at CE of 35 ± 17 V over *m*/*z* 10–800. Precursor ions, fragment ions, and the corresponding formulas are listed in the supplementary information (Table S1).

### Glucuronidase treatment

The urine (50 μL) was mixed with 5 μL of 0.1 mol/L phosphate buffer (pH 6). Then, 5 μL of 1 mg/mL glucuronidase in 10 mM phosphate buffer was added and the mixture was incubated at 37 °C for 3 h. To this, 0.1% formic acid-acetonitrile (100 μL) was added and the mixture was centrifuged (6800×*g*, 5 min). The supernatant was evaporated under the stream of nitrogen at 40 °C. Methanol (25 μL) and water (25 μL) were added to the residue in turn, and the mixture was stirred vigorously and centrifuged (6800×*g*, 5 min). The supernatant was transferred to a vial. An aliquot (10 μL) was injected into the LC-QqQMS system. At the same time, urine extract without glucuronidase treatment (5 μL of 10 mM buffer was added instead of the glucuronidase solution) was also prepared. The urine was extracted and analyzed in triplicate and averages of peak areas were used to evaluate the changes caused by glucuronidase treatment (Table S2 in the supplementary information).

## Result

### Synthesis of hydroxyzolpidems

**Fig. 2:**
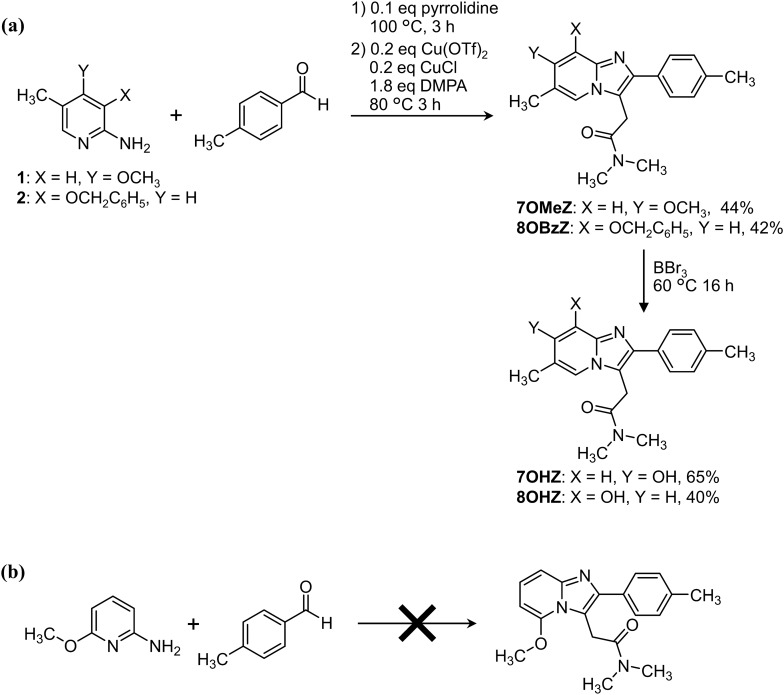
**a** Synthesis of 7OHZ and 8OHZ. **b** Attempt to synthesize a 5-methoxyzolpidem analogue

7OMeZ and 8OBzZ were synthesized via the three-component coupling reaction, which was developed in our previous study [9]. The yields were 44% and 42%, for 7OMeZ and 8OBzZ, respectively (Fig. 2a). Demethylation of 7OMeZ and debenzylation of 8OBzZ with BBr_3_ afforded 7OHZ and 8OHZ in yields of 65% and 40%, respectively. The ^1^H and ^13^C NMR spectra of 7OHZ hydrobromide and 8OHZ tosylate are shown in the supplementary information. It seemed that 7OHZ was not stable in non-ionic form in solution; the solution of neutralized 7OHZ gradually darkened within hours. However, the acid salts of 7OHZ, such as hydrobromide, were stable even in solution at least for months at − 20 °C.

We attempted to synthesize 5-hydroxyzolpidem similarly via the same coupling reaction using 6-alkoxy-2-amino-5-methylpyridine, followed by the dealkylation. However, the precursor material was not commercially available. Before starting to synthesize the precursor material, we attempted the coupling reaction with commercially available 2-amino-6-methoxypyridine, in which the methyl group on the pyridine ring was absent. However, the reaction did not afford the desired product, 6-desmethyl-5-methoxyzolpidem (Fig. 2b). Therefore, we did not further attempt to synthesize 5-hydroxyzolpidem. This result demonstrated the limitation of the coupling reaction; the alkoxy group at the 6-position of 2-aminopyridine inhibits the reaction.

### LC-QqQMS analysis of 7OHZ, and 8OHZ

The product ion spectra of 7OHZ and 8OHZ with various CEs (15, 35, 55, and 75 V) are shown in the supplementary information (Fig. S1). They were almost identical at 15 and 35 V; fragments at *m*/*z* 279 and 251 were assigned to the loss of NMe_2_ and NMe_2_CO, respectively. At 55 and 75 V, fragments that are attributed to the hydroxy-methylpyridine (*m*/*z* 108) were observed in both 7OHZ and 8OHZ. All product ion spectra of ZOL and its metabolites synthesized in our laboratory (M1–M4, 7OHZ, and 8OHZ) showed the same fragmentation patterns: (a) loss of NMe_2_ (− 45 Da) and (b) loss of NMe_2_CO (− 73 Da), and (c) dissociation of the pyridine moiety (*m*/*z* 92 for ZOL, M1, and M3; *m*/*z* 108 for M4, 7OHZ, and 8OHZ; and *m*/*z* 122 for M2) at 55 and 75 V.

**Fig. 3:**
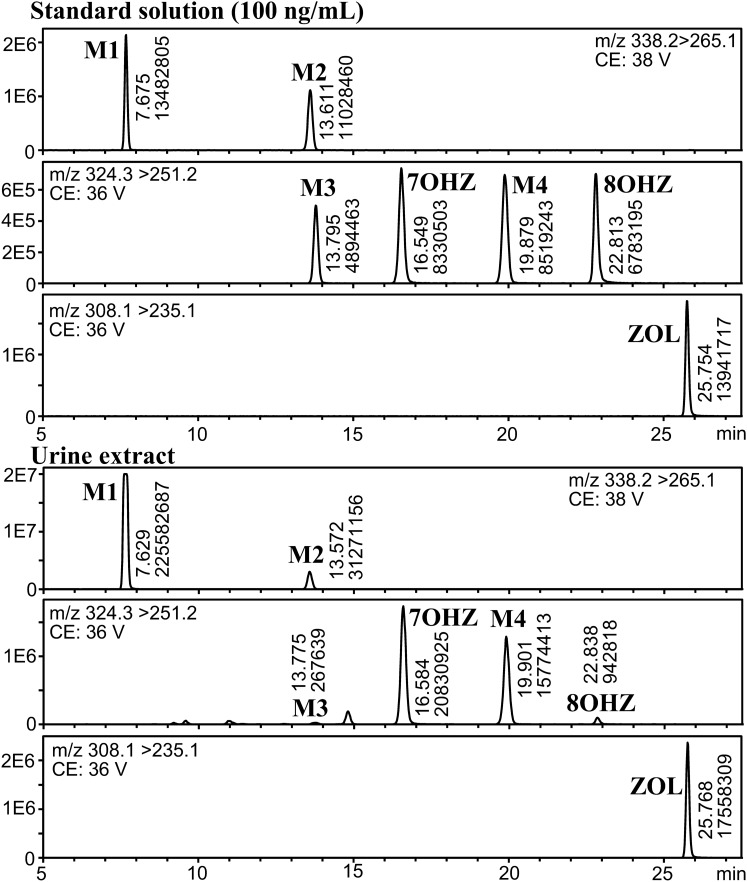
Mass chromatograms of synthetic standards at a concentration of 100 ng/mL (above) and urine extract (below)

The chromatograms obtained from the analysis of 7OHZ and 8OHZ along with ZOL and previously synthesized M1–M4 at concentrations of 100 ng/mL are shown in Fig. 3. The hydroxyzolpidems were clearly separated and eluted in the order of M3, 7OHZ, M4, and 8OHZ using the octadecyl silica (C18) column with gradient elution of methanol − 10 mM aqueous ammonium formate.

The extract of the urine specimen was analyzed under the same conditions, and the mass chromatograms are also shown in Fig. 3. The most intense peak among hydroxyzolpidems was assigned to 7OHZ. The presence of 8OHZ was also confirmed, but its peak intensity was much lower. Peak intensities of hydroxyzolpidems were in the order of 7OHZ > M4 >> 8OHZ > M3.

### Search for other ZOL metabolites

**Fig. 4:**
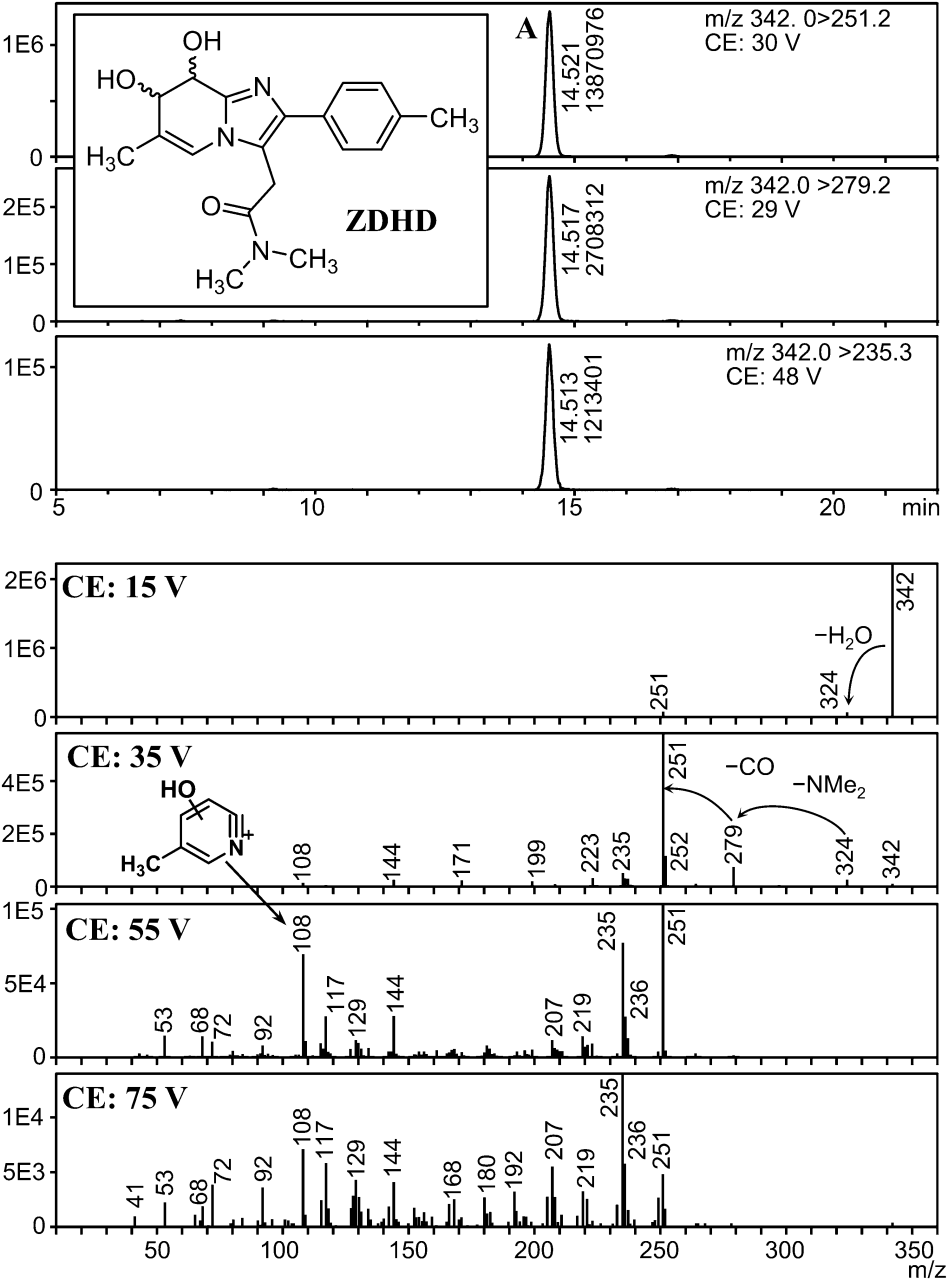
Mass chromatograms (above) and mass spectra (below) of peak A

**Fig. 5:**
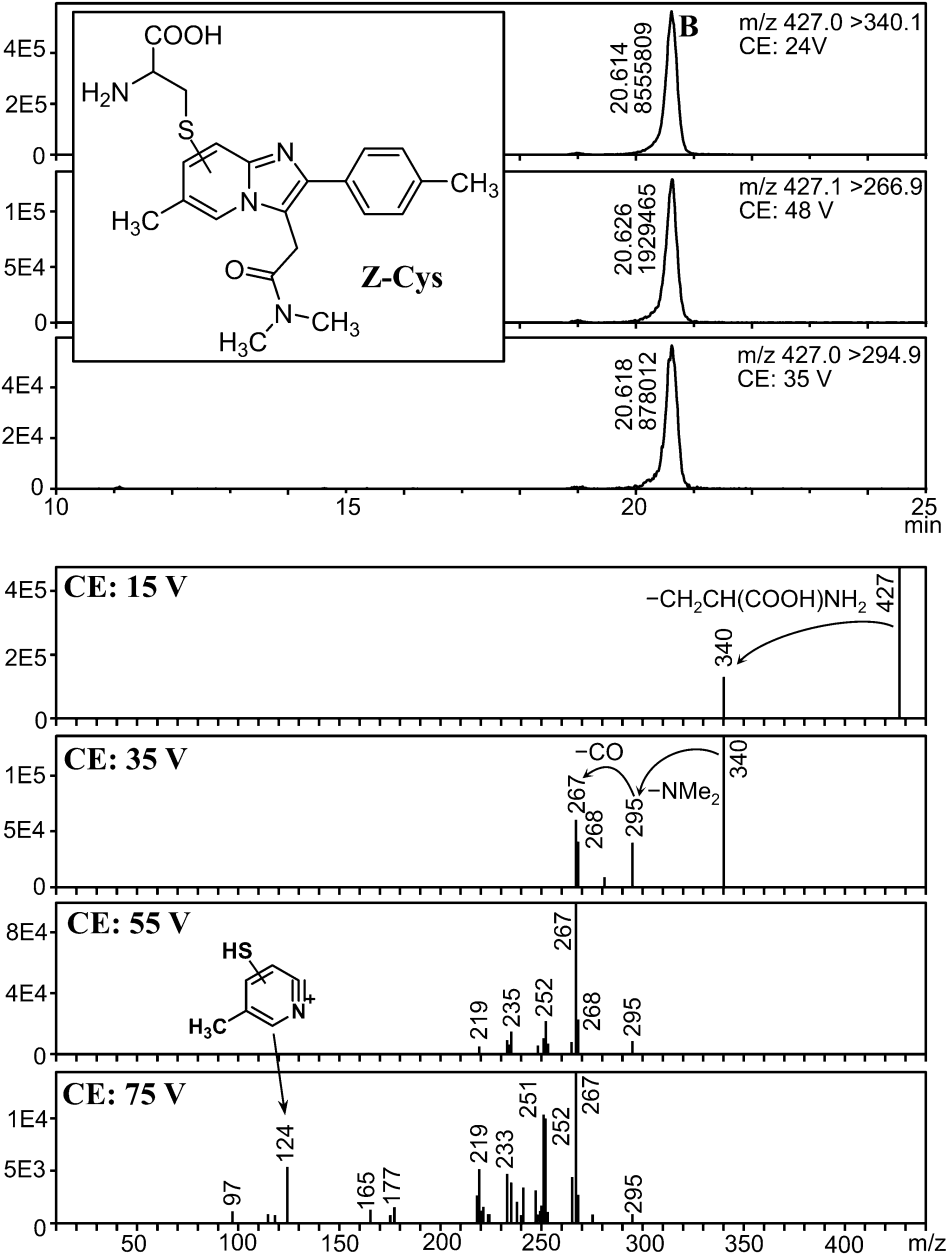
Mass chromatograms (above) and mass spectra (below) of peak B

**Fig. 6:**
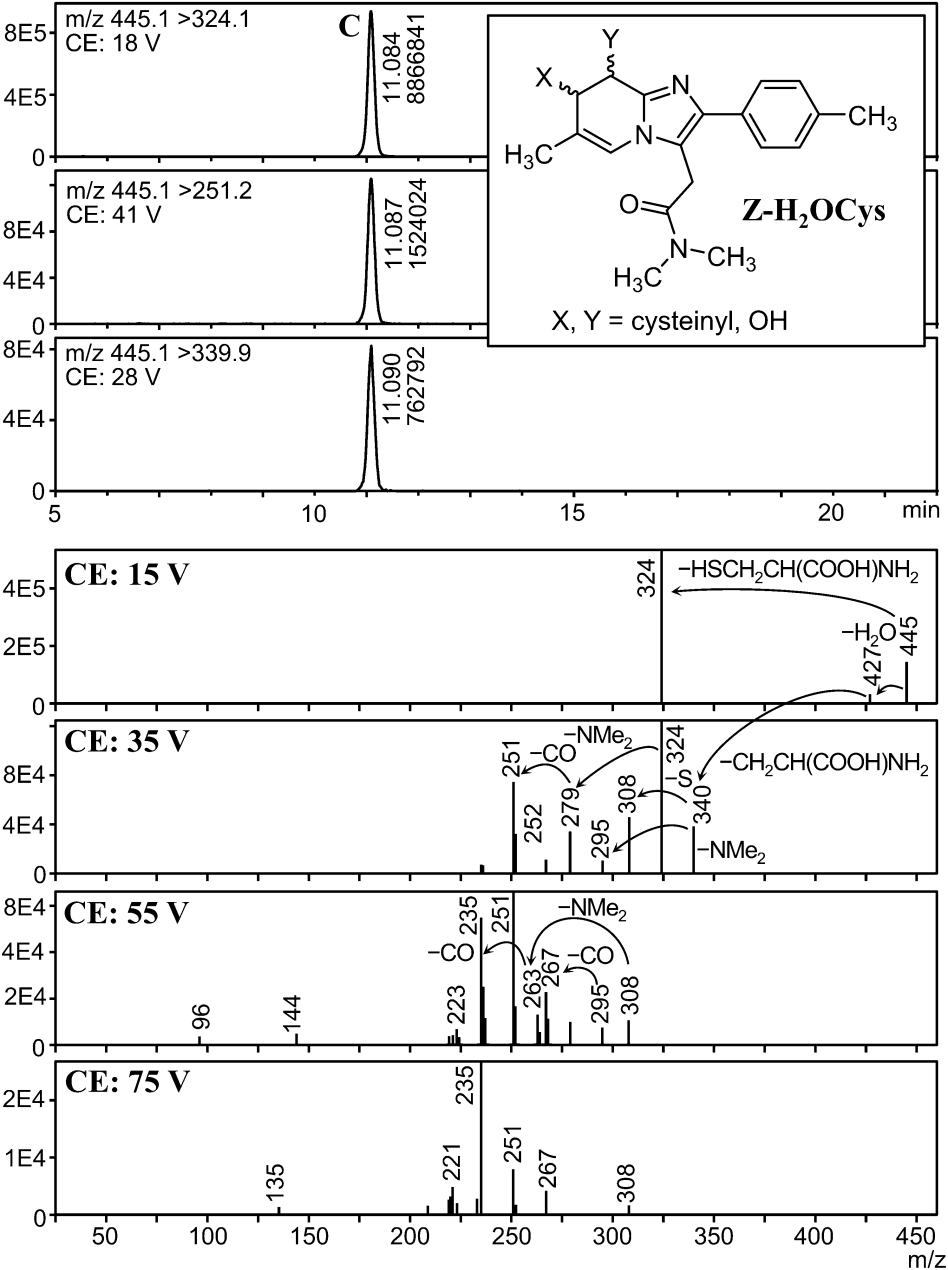
Mass chromatograms (above) and mass spectra (below) of peak C

**Fig. 7:**
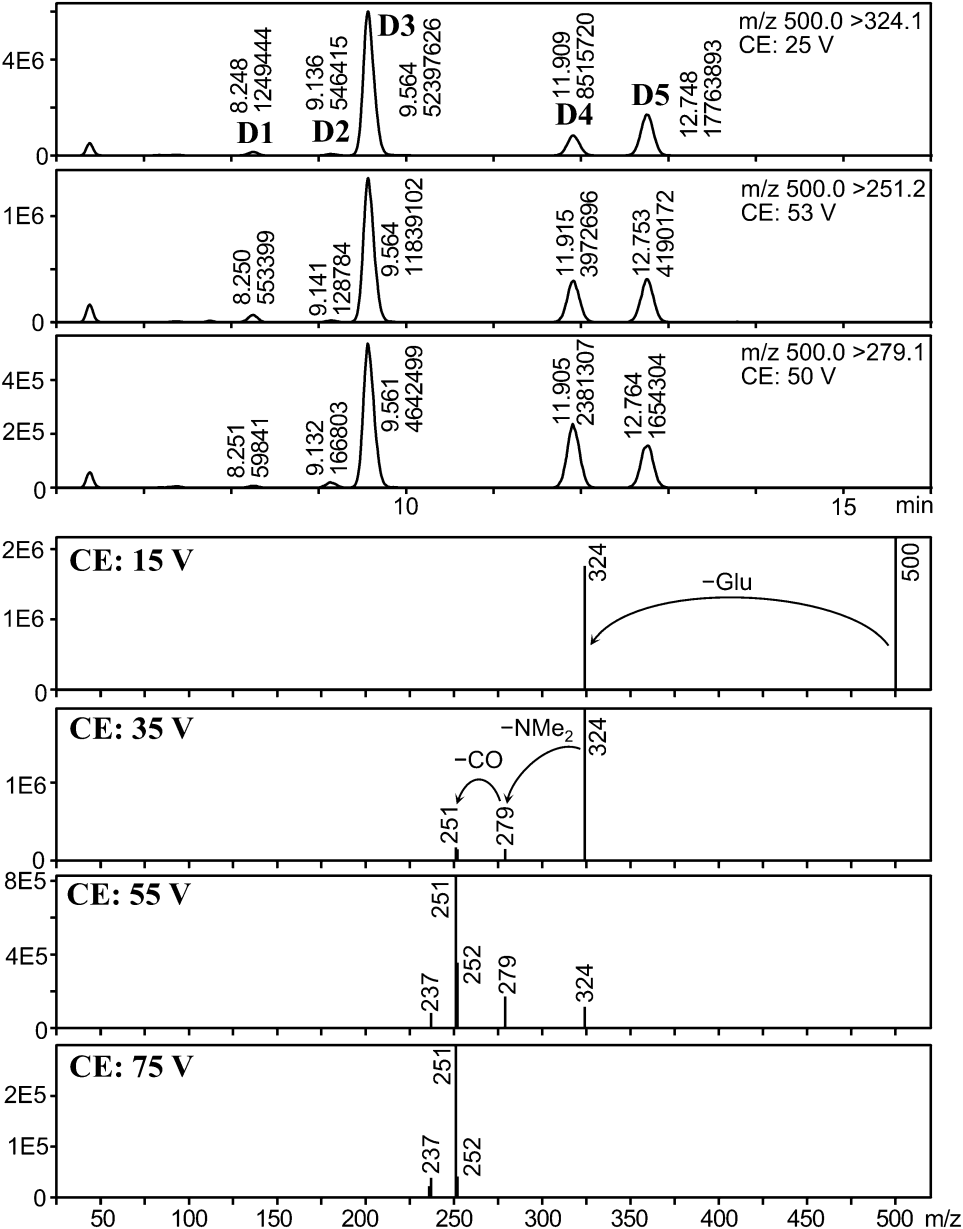
Mass chromatograms of peak D1–D5 (above) and mass spectra of peak D3 (below)

**Fig. 8:**
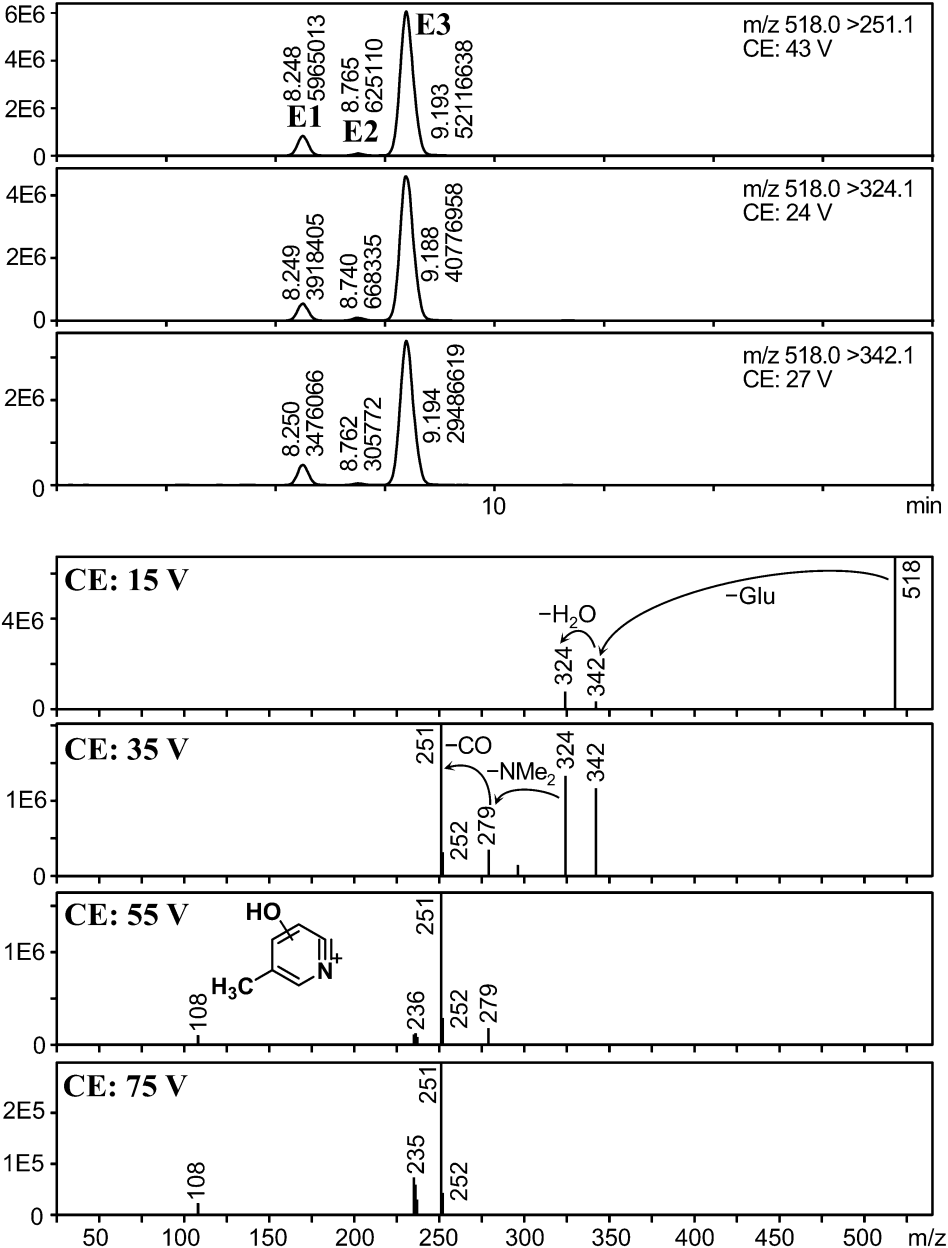
Mass chromatograms of peak E1–E3 (above) and mass spectra (below) of peak E3

We found five peaks with a fragmentation pattern similar to that of ZOL, and their precursor ions were *m*/*z* 342, 427, 445, 500, and 518 (peaks A, B, C, D, and E, respectively). To reveal the presence of isomers for each metabolite, analysis with higher sensitivity was necessary. Therefore, MRM parameters for these metabolites were optimized using the urine extract, and analyzed the extract again in MRM mode. Mass chromatograms and product ion spectra of these peaks at various CEs are shown in Figs. 4, 5, 6, 7 and 8.

Peak A appeared at 14.5 min (Fig. 4). The precursor ion was *m*/*z* 342, which corresponds to “[ZOL + H]^+^ + H_2_O_2_”. In its product ion spectra at 15 V, a fragment was observed at *m*/*z* 324, indicating a facile loss of H_2_O. Fragments of *m*/*z* 279 and 251 were assigned to loss of NMe_2_ and NMe_2_CO from the fragment of *m*/*z* 324, respectively. The *m*/*z* values of fragments observed at 55 and 75 V (*m*/*z* 235, 207, 144, 129, 108, 68, and 53) were identical to those observed in the product ion spectrum of 8OHZ. These results indicate that this compound is a hydrated form of 8OHZ. The results from LC-QqTOFMS analysis reinforced these assignments; the *m*/*z* value of peak A (*m*/*z* 342.1813, Table S1) corresponds to [ZOL + H]^+^ + H_2_O_2_, and the fragments at *m*/*z* 324.1705, 279.1134, and 251.1180 corresponded to the fragments described above. Considering that this compound was formed by ZOL metabolism, the most plausible structure of peak A is ZOL dihydrodiol (ZDHD), which has two hydroxy groups at the 7- and 8-positions and a saturated C–C bond between them, as depicted in Fig. 4. There are two possible isomers, *anti*-7,8-diol and *syn*-7,8-diol, but the stereochemistry cannot be elucidated by mass spectrometry alone.

Peak B (precursor ion: *m*/*z* 427) appeared at 20.6 min (Fig. 5). Its product ion spectra show a facile loss of 87 Da that afforded a fragment at *m*/*z* 340, followed by the loss of NMe_2_ (*m*/*z* 295) and NMe_2_CO (*m*/*z* 267). These fragments were 32 Da higher than those of ZOL. In addition, a fragment at *m*/*z* 124 was observed at 75 V, which indicated that “32 Da” was bound to the pyridine ring. The result of LC-QqTOFMS analysis revealed that the “32 Da” corresponds to one sulfur atom and the formula of this metabolite is “ZOL + C_3_H_5_NO_2_S”. From these observations, peak B can plausibly be attributed to a cysteine adduct of ZOL (Z-Cys, Fig. 5). The cysteine is bound somewhere on the pyridine ring, but its exact location could not be elucidated.

Peak C (precursor ion: *m*/*z* 445) appeared at 11.1 min (Fig. 6). Its product ion spectra revealed three fragmentation pathways. (i) A loss of water (*m*/*z* 427) at 15 V, followed by the formation of sulfur-bound ZOL (*m*/*z* 340, 295, and 267). This pathway is identical to that of Z-Cys. (ii) A loss of cysteine (*m*/*z* 324) at 15 V, followed by the same fragmentation as those of hydroxyzolpidems (*m*/*z* 279 and 251), and (iii) formation of ZOL (*m*/*z* 308, 263, and 235). The result of LC-QqTOFMS analysis reinforced these assignments. From these results, this metabolite is assigned to the hydrated form of Z-Cys, that is, the cysteine adduct of dihydro(hydroxy)zolpidem (Z-H_2_OCys), as depicted in Fig. 6. The exact location of the cysteine and hydroxy group, including the stereochemistry is unknown.

Mass chromatograms of peak D1–D5 (precursor ion: *m*/*z* 500), and product ion spectra of the most intense peak (D3) are shown in Fig. 7. The product ion spectra indicate loss of glucuronic acid (− 176 Da, *m*/*z* 324), and formation of the fragments identical to those of hydroxyzolpidems (*m*/*z* 279 and 251). In addition, the peaks D1–D5 disappeared after glucuronidase treatment, and the peak areas of the four hydroxyzolpidems (M3, M4, 7OHZ, and 8OHZ) increased (Table S2). From these results, peaks D1–D5 were assigned to glucuronides of M3, M4, 7OHZ, 8OHZ, and one more unidentified hydroxyzolpidem.

We found one more group of glucuronides. In Fig. 8, three peaks were observed on the mass chromatograms (precursor ion: *m*/*z* 518, peak E1, E2, and E3). In addition, the product ion spectra of the most intense peak (E3) showed loss of glucuronide (*m*/*z* 342), and glucuronide and water (*m*/*z* 324) at 15 V. After glucuronidase treatment, peaks E1, E2, and E3 almost disappeared (peak areas less than 0.3%), whereas the peak areas of ZDHD increased by 7.6-fold (Table S2). From these results, peak E1–E3 were assigned to glucuronides of ZDHD (ZDHD-Glus). The presence of three peaks indicates ZDHD-Glus has at least three isomers. This can be explained by the presence of the two hydroxy groups and the stereochemistry of ZDHD: there are two possible isomers (*syn* and *anti*) and two enantiomers for each. For example, if ZDHD has *anti* geometry, ZDHD consists of two enantiomers, (7R, 8R)- and (7S, 8S)-ZDHD, which are not separable without chiral chromatography, and glucuronidation of *anti*-ZDHD affords four structural isomers, (7R, 8R)-ZDHD-7-glucuronide, (7R, 8R)-ZDHD-8-glucuronide, (7S, 8S)-ZDHD-7-glucuronide, and (7S, 8S)-ZDHD-8-glucuronide.

## Discussion

### Synthesis and analysis of 7OHZ and 8OHZ

We have successfully synthesized four hydroxyzolpidems (M3, M4, 7OHZ, and 8OHZ) so far. Their product ion spectra were almost identical, meaning that chromatographic separation was necessary. Their peaks were clearly separated by the optimized conditions in this study and were successfully assigned as a result.

The presence of 7OHZ and 8OHZ in human urine obtained from the deceased was confirmed, and the peak intensity of 7OHZ was the highest among the hydroxyzolpidems. The presence of the hydroxyzolpidem with a hydroxy group on the pyridine ring was first reported by Thénot et al. [10], but the exact location of the hydroxy group was not determined. The structure of this metabolite was identified as 7OHZ for the first time in the present study by comparison with synthetic standards. Thénot et al. also reported that the ratio of the metabolites eliminated in human urine and feces were 51.5%, 11.5%, and 10.1% for M1, M2, and “hydroxylation on the imidazopyridine ring”, respectively. In the present study, the peak intensity of M1 was strongest, that of M2 was the second highest, and 7OHZ followed. These results suggest that the ratio of metabolites is approximately same between the previous study and this study.

### Search for novel metabolites and proposal of novel metabolic pathways

Many studies have focused on the analysis of ZOL metabolites in human body fluids [3, 10–15], but to our knowledge, ZDHD, Z-Cys, and Z-H_2_OCys were found for the first time in this study. We analyzed the urine sample obtained from a deceased man who was elderly (in his eighties) and had been prescribed ZOL 2 weeks before his death. The clearance of ZOL is reported to be lower in the elderly than in young adults, resulting in higher maximum blood concentration and longer elimination half-life [16]. Therefore, ZOL had likely stayed in the deceased’s body longer than it would have in a young adult, which resulted in the accumulation of minor metabolites, such as ZDHD, Z-Cys, and Z-H_2_OCys.

Concerning glucuronides, Rossi et al. investigated urinary metabolites after intake of 10 mg ZOL and found that hydroxyzolpidem and ZOL carboxylic acids are partially glucuroconjugated [11]. In our study, we revealed the presence of glucuronides of hydroxyzolpidems, which agreed with Rossi’s result, whereas we could not find any acyl glucuronides. Our result from the glucuronidase treatment experiment also indicated that glucuronides of M1 and M2 were not present in the sample. The acyl glucuronides of M1 and M2 probably decomposed during the time between death and autopsy, because acyl glucuronides are known to be instable metabolites and easily hydrolyzed to carboxylic acids [17].

**Fig. 9:**
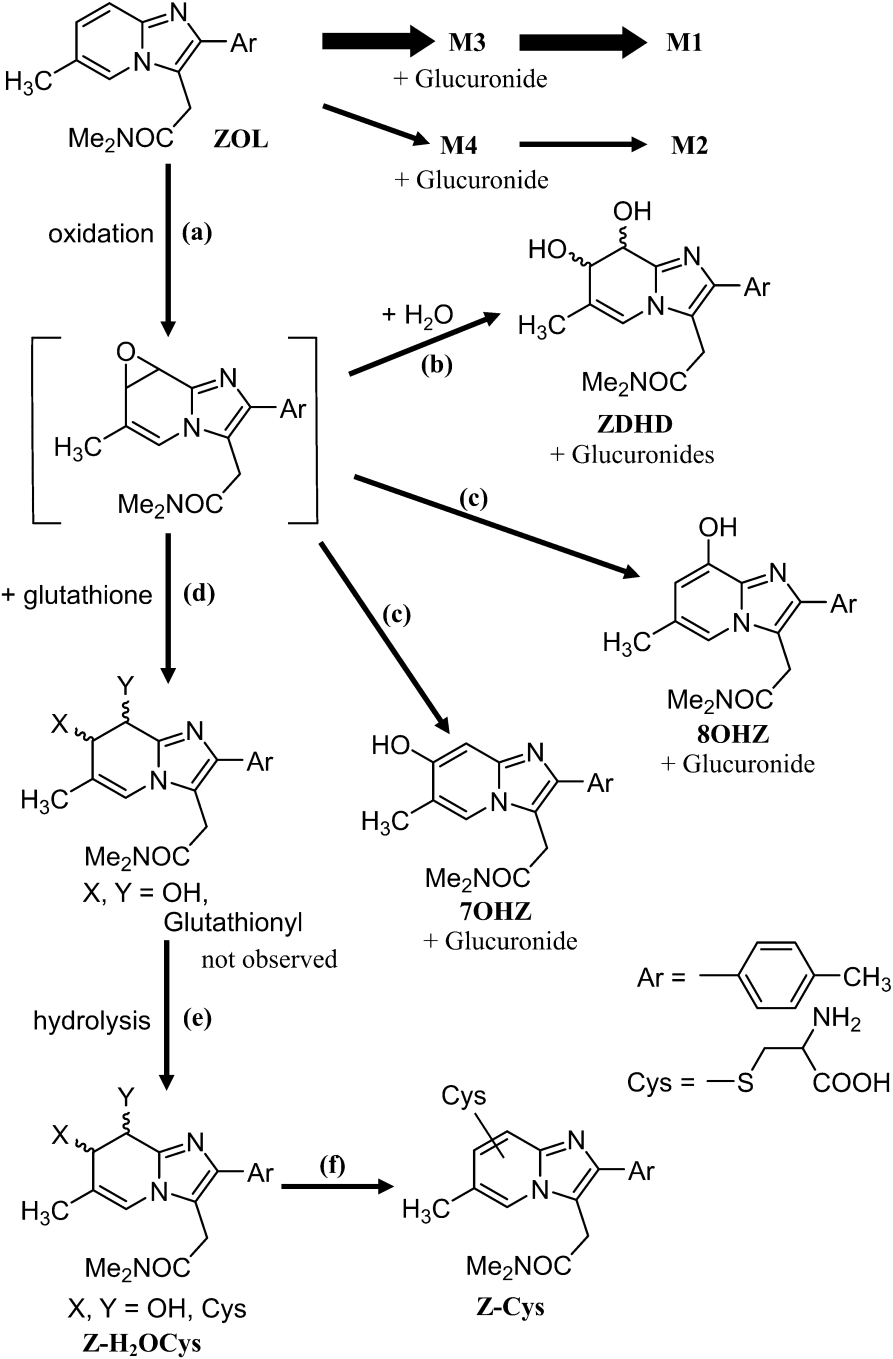
Proposed metabolic pathway of ZOL based on the findings in this study

To postulate a novel metabolic pathway, we referred to the metabolism of alpidem, which has the same imidazo[1,2-*α*]pyridine structure [8]. The metabolic pathway of alpidem includes the formation of an epoxide at C–C bond between the 7- and 8-position as an intermediate. The epoxide reacts with glutathione to afford the glutathione adduct of dihydro(hydroxy)alpidem, followed by dehydration that affords glutathione adducts of alpidem. We present new metabolic pathways of ZOL, which forms ZDHD, Z-Cys, Z-H_2_OCys, 7OHZ, and 8OHZ, as illustrated in Fig. 9. First, oxidation at the C–C double bond between the 7- and 8-positions forms ZOL epoxide as an intermediate (a). Reaction of the epoxide with water affords ZDHD (b). Proton migration in the epoxide affords 7OHZ and 8 OHZ (c). Reaction of the epoxide with glutathione affords glutathione adduct of dihydro(hydroxy)zolpidem, which was not detected in this study (d). Elimination of amino acids from the glutathione adduct forms Z-H_2_OCys (e). Dehydration from Z-H_2_OCys affords Z-Cys (f). We could not find the glutathione adduct of dihydro(hydroxy)zolpidem, which is the key metabolite in this pathway. This was probably because this metabolite is not eliminated in urine before further metabolism proceeds.

Alpidem was withdrawn from the market worldwide, because it causes severe liver damage [18]. One of the reasons for this is that the epoxide intermediate acts as a reactive metabolite, which was supported by the results showing that a high concentration of alpidem (500 μM) in isolated rat hepatocytes induces glutathione depletion, whereas the same concentration of ZOL causes only moderate glutathione depletion [19]. The results of the present study indicate that there is a metabolic pathway of ZOL that consumes glutathione, but the pathway involves only a small fraction of ZOL administered.

As reported in many studies, the major metabolite of ZOL in urine is M1. Detection of ZOL and M1 is enough proof for ZOL misuse. However, this study revealed the presence of several novel metabolites in urine that are detectable using LC–MS/MS. These metabolites are formed from a small fraction of ZOL intake but are expected to show different characteristics in the excretion and distribution in human body; therefore, further studies into these metabolites from a toxicological perspective are awaited, for example, quantification of these metabolites in urine obtained from various type of subjects (age, sex, etc.). We are currently trying to develop a method for simultaneously quantifying ZOL, M1–M4, 7OHZ, and 8OHZ in urine, including validation of the method and its application to autopsy samples.

## Conclusions

We successfully synthesized 7OHZ and 8OHZ, and their presence in postmortem urine was confirmed. The peak intensity of M1 was predominant, and those of hydroxyzolpidems were in the order of 7OHZ > M4 >> 8OHZ > M3. We also searched for other urinary ZOL metabolites and found ZDHD and its glucuronides (ZDHD-Glus), Z-Cys, Z-H_2_OCys, and Z-OGlus. The presence of these metabolites led to identification of novel metabolic pathways, including epoxide formation between the 7- and 8-positions as an intermediate.

## Supplementary Information

Below is the link to the electronic supplementary material.

Supplementary file1 (PDF 156 kb)

Supplementary file2 (PDF 389 kb)

Supplementary file3 (PDF 7104 kb)
