# Supplementary material for: Qualitative analysis of 7- and 8-hydroxyzolpidem and discovery of novel zolpidem metabolites in postmortem urine using liquid chromatography–tandem mass spectrometry
Source: Forensic Toxicol. 2022 Jan 4;40(2):263–77. doi: 10.1007/s11419-021-00611-9 (PMC9715527; doi:10.1007/s11419-021-00611-9)
Supplement: Supplementary file 1 — Supplementary file1 (PDF 156 kb) [file 11419_2021_611_MOESM1_ESM.pdf]

Qualitative analysis of 7- and 8-hydroxyzolpidem and discovery of novel zolpidem metabolites in postmortem urine using liquid chromatography-tandem mass spectrometry

Koji Yamaguchi[1] · Hajime Miyaguchi[2] · Youkichi Ohno[1] · Yoshimasa Kanawaku[1]

Supplementary Information: Synthesis of 2-amino-4-methoxy-5-methylpyridine (1) and 2-amino-3-benzyloxy-5-methylpyridine (2)

2-Aminopyridine derivatives **1** and **2** were synthesized following the scheme depicted in Figs. S2 and S3, respectively.

Reagents

2-Bromo-5-methylpyridine, 2-amino-3-bromo-5-methylpyridine, and 3,4,7,8-tetramethyl-1,10-phenanthroline (Me_4Phen) were purchased from Tokyo Chemical Industry Co., Ltd. (Tokyo, Japan). Copper(I) iodide (CuI) and NH_2 silica gel (Wakogel, 50NH2) were purchased from Fujifilm Wako Pure Chemical Co. (Osaka, Japan). Other chemicals were purchased from Kanto Chemical Co., Inc. (Tokyo, Japan).

Nuclear magnetic resonance spectroscopy

^1H and ^{13}C nuclear magnetic resonance (NMR) spectra were recorded using an Avance III HD 400 (Brucker Co., Billerica, MA) or ECA-600 (JEOL Ltd., Tokyo, Japan) spectrometer. Chemical shifts were referenced to the tetramethylsilane (TMS) signal at $\delta = 0$ ppm in chloroform-*d* ($CDCl_3$).

High-resolution mass spectrometry

High-resolution mass spectrometry (HR-MS) measurements were performed using a Q Exactive mass spectrometer (Thermo Fisher Scientific, Waltham, MA) with an electrospray ionization (ESI) probe. Full scan mass spectra were recorded in positive mode. The conditions for

ESI were as follows: sheath gas flow rate, 5 units; capillary temperature, 320 °C; spray voltage, 3.5 kV; probe heater temperature, 350 °C; S-lens RF level, 50 units. Products were dissolved in a 1:1 (*v*/*v*) mixture of water–acetonitrile containing 0.1% formic acid at a concentration of 1 μg/mL and infused into the spectrometer at a flow rate of 10 μL/min.

*1. Synthesis of 2-bromo-5-methylpyridine N-oxide (**3**)*

2-Bromo-5-methylpyridine (3.45 g, 20.0 mmol) was dissolved with chloroform (50 mL), followed by addition of *m*-chloroperoxybenzoic acid (mCPBA, 6.86 g, 30.6 mmol). The mixture was stirred at room temperature for 4 days. Saturated aqueous sodium hydrogen sulfite solution (3 mL) was added and stirred for 15 min. Aqueous sodium carbonate (10% [*v*/*v*], 50 mL) was added and the mixture was transferred to a separation funnel. The organic layer was separated, and the aqueous phase was extracted with chloroform (50 mL). The organic extracts were combined, dried with anhydrous sodium sulfate, and evaporated to dryness. The residue was dissolved with ethyl acetate (10 mL) and passed through a NH_2 silica gel column (3cm [i.d.] × 1 cm [height]). The NH_2 silica gel was washed with ethyl acetate (60 mL). The eluate was concentrated to approximately 5 mL and diisopropyl ether (20 mL) was added. White crystals were formed, which were separated and dried in vacuo. Yield: 3.18 g (16.9 mmol, 84%). ^{1}H NMR (400 MHz, $CDCl_3$) δ 2.30 (s, 3H), 6.96 (d, *J* = 8.23 Hz, 1H), 7.54 (d, *J* = 8.23 Hz, 1H), 8.26 (s, 1H). ^{13}C NMR (100 MHz, $CDCl_3$) δ 18.00, 127.33, 129.73, 130.03, 135.60, 140.35. Elemental analysis calcd for C_6H_6BrNO: C 38.33%, H 3.22%, N 7.45%; found: C 38.20%, H 3.36%, N 7.18%. HR-MS: calcd for C_6H_7BrNO ($[M+H]^+$) 187.9706; found 187.9707.

*2. Synthesis of 2-bromo-4-nitro-5-methylpyridine N-oxide (**4**)*

Compound **3** (3.76 g, 20.0 mmol) was dissolved in sulfuric acid (5 mL). Nitric acid (5 mL) was added to this solution. The mixture was carefully heated to 100 °C and stirred for 2 h. The mixture was cooled to room temperature, and crushed ice (40 g) was added. Saturated aqueous sodium carbonate was added until the generation of CO_2 ceased. The reaction mixture was extracted three

times with chloroform (50 mL). Organic phases were combined, dried with sodium sulfate, and evaporated to dryness. The residue was dissolved with ethyl acetate (50 mL) and passed through a NH_2 silica gel column (3 cm [i.d.] × 1 cm [height]). The column was washed with ethyl acetate (50 mL). The eluate was concentrated under reduced pressure and recrystallization from ethyl acetate–diisopropyl ether afforded compound **4** as pale-yellow crystals. Yield: 2.74 g (11.8 mmol, 59%). ^{1}H NMR (400 MHz, $CDCl_3$) δ 2.59 (s, 3H), 8.30 (s, 1H), 8.41 (s, 1H). ^{13}C NMR (100 MHz, $CDCl_3$) δ 17.95, 126.16, 130.70, 131.08, 142.03, 142.30. Elemental analysis calcd for $C_6H_5BrN_2O_3$: C 30.93%, H 2.16%, N 12.02%; found: C 30.87%, H 2.28%, N 11.81%. HR-MS: calcd for $C_6H_6BrN_2O_3$ ($[M+H]^+$) 232.9556; found 232.9556.

*3. Synthesis of 2,4-dibromo-5-methylpyridine N-oxide (**5**)*

Compound **4** (2.33 g, 10.0 mmol) was dissolved in acetic acid (20 mL) and acetyl bromide (8.04 g, 65.4 mmol) was added. The mixture was heated at 80 °C for 90 min, and then evaporated to dryness. An aqueous solution of Na_2CO_3 (20% [*v*/*w*], 50 mL) was added to the residue, and the solution was extracted with chloroform (30 mL) three times. The extract was dried with sodium sulfate and evaporated to dryness. The residue was dissolved in ethyl acetate (30 mL) and passed through a NH_2 silica gel column (3 cm [i.d.] × 1 cm [height]). The column was washed with ethyl acetate (50 mL). The eluate was concentrated under reduced pressure and recrystallization from ethyl acetate afforded **5** as white crystals. Yield: 2.15 g (8.05 mmol, 80%). ^{1}H NMR (400 MHz, $CDCl_3$) δ 2.31 (s, 3H), 7.81 (s, 1H), 8.26 (s, 1H). ^{13}C NMR (100 MHz, $CDCl_3$) δ 19.64, 121.35, 130.45, 132.77, 135.90, 140.16. Elemental analysis calcd for $C_6H_5Br_2NO$: C 27.00%, H 1.89%, N 5.25%; found: C 26.94%, H 1.97%, N 4.99%. HR-MS: calcd for $C_6H_6Br_2NO$ ($[M+H]^+$) 265.8811; found 265.8812.

*4. Synthesis of 2-benzylamino-4-bromo-5-methylpyridine N-oxide (**6**)*

A mixture of **5** (2.66 g, 9.98 mmol) and benzylamine (3.94 g, 36.9 mmol) was heated at 100 °C overnight. After cooling to room temperature, 20% (*v*/*w*) aqueous solution of Na_2CO_3 (50 mL) was

added and the solution was extracted three times with chloroform (30 mL). The extract was dried with sodium sulfate and evaporated to dryness. The residue was dissolved in ethyl acetate (100 mL) and passed through a NH_2 silica gel column (4 cm [i.d.] × 2 cm [height]). The column was washed with ethyl acetate (60 mL). The eluate was concentrated by evaporation under reduced pressure, and recrystallization from ethyl acetate–diisopropyl ether afforded **6** as white crystals. Yield: 2.23 g (7.61 mmol, 76%). ^{1}H NMR (400 MHz, $CDCl_3$) δ 2.21 (s, 3H), 4.47 (d, *J* = 5.99 Hz, 2H), 6.74 (s, 1H), 7.06 (brt, *J* = 5.99 Hz, 1H), 7.2–7.4 (m, 5H), 8.01 (s, 1H). ^{13}C NMR (100 MHz, $CDCl_3$) δ 18.88, 46.28, 108.93, 121.85, 124.75, 127.04, 127.85, 128.91, 136.68, 137.11, 148.95. Elemental analysis calcd for $C_{13}H_{13}BrN_2O$: C 53.26%, H 4.47%, N 9.56%; found: C 53.11%, H 4.34%, N 9.26%. HR-MS: calcd for $C_{13}H_{14}BrN_2O$ ($[M+H]^+$) 293.0284; found 293.0282.

*5. Synthesis of 2-benzylamino-4-methoxy-5-methylpyridine (**7**)*

A mixture of **6** (1.17 g, 4.00 mmol), K_2CO_3 (1.16 g, 8.39 mmol), and methanol (20 mL) was added to a 50 mL screw vial. The vial was sealed and heated at 100 °C for 3 days. The mixture was cooled to room temperature, filtrated, and evaporated to dryness. Acetic acid (10 mL), water (10 mL), and ion powder (1.03 g 18.5 mmol) were added to the residue, and the mixture was heated at 100 °C for 90 min. After cooling to room temperature, the mixture was basified with 10% (*v*/*w*) aqueous sodium carbonate. Extraction with ethyl acetate (20 mL) was repeated until all of the target compound was extracted (confirmed by thin layer chromatography). The extract was dried with sodium sulfate and concentrated to approximately 20 mL under reduced pressure. The residue was passed through a silica gel column (3 cm [i.d.] × 2 cm [height]) and the column was washed with ethyl acetate (30 mL). The eluate was evaporated to dryness. Recrystallization from diisopropyl ether afforded **7** as white crystals. Yield: 0.544 g (60%). ^{1}H NMR (400 MHz, $CDCl_3$) δ 2.02 (s, 3H), 3.74 (s, 3H), 4.49 (d, *J* = 5.81 Hz, 2H), 4.78 (brt, *J* = 5.81 Hz, 1H), 5.82 (s, 1H), 7.2-7.4 (m, 5H), 7.74 (s, 1H). ^{13}C NMR (100 MHz, $CDCl_3$) δ 12.43, 46.68, 54.76, 88.35, 112.51, 127.17, 127.44, 128.60, 139.48, 148.22, 159.29, 165.43. Elemental analysis calcd for $C_{14}H_{16}N_2O$: C 73.66%, H 7.06%, N 12.27%; found: C 73.40%, H 6.93%, N 12.09%. HR-MS: calcd for $C_{14}H_{17}N_2O$ ($[M+H]^+$)

229.1335; found 229.1333.

*6. Synthesis of 2-amino-4-methoxy-5-methylpyridine (**1**)*

Compound **7** (115 mg, 0.502 mmol) was added to sulfuric acid (1 mL), and the mixture was stirred and heated at 40 °C for 90 min. After cooling to room temperature, crushed ice (5 g) was added to the mixture. Saturated aqueous sodium carbonate was added until the evolution of carbon dioxide ceased. Water (10 mL) was added and extracted three times with ethyl acetate (20 mL). Organic phases were combined, dried with sodium sulfate, and evaporated to dryness. Recrystallization from ethyl acetate–diisopropyl ether afforded **1** as white crystals. Yield: 52.2 mg (75%). ^{1}H NMR (400 MHz, $CDCl_3$) δ 2.03 (s, 3H), 3.81 (s, 3H), 4.29 (brs, 2H), 5.97 (s, 1H), 7.70 (s, 1H). ^{13}C NMR (100 MHz, $CDCl_3$) δ 12.45, 54.89, 90.14, 113.41, 148.10, 158.81, 165.56. Elemental analysis calcd for $C_7H_{10}N_2O$: C 60.85%, H 7.30%, N 20.28%; found: C 60.71%, H 7.10%, N 20.28%. HR-MS: calcd for $C_7H_{11}N_2O$ ($[M+H]^+$) 139.0866; found 139.0864.

*Synthesis of 2-amino-3-benzyloxy-5-methylpyridine (**2**)*

2-Amino-3-bromo-5-methylpyridine (2.62 g, 14.02 mmol), cesium carbonate (6.91 g, 21.21 mmol), Me_4Phen (0.502 g, 2.13 mmol), CuI (0.272 g, 1.43 mmol), and benzyl alcohol (5.25 g, 48.59 mmol) were added to a glass vial and heated at 90 °C for 17 h. The reaction mixture was mixed with ethyl acetate (20 mL) and passed through a NH_2 silica gel column (9 cm [i.d.] × 1 cm [height]). The column was washed with ethyl acetate (100 mL). The eluate was extracted four times with 1% (*v*/*v*) hydrochloric acid(50 mL). The aqueous extract was basified with sodium carbonate and extracted four times with ethyl acetate (50 mL). The extract was evaporated to dryness. The desired product (**2**) was purified by column chromatography (silica gel, eluent: 1:1 ethyl acetate–hexane) and recrystallization from diisopropyl ether as white crystals. Yield: 2.08 g (9.71 mmol, 69%). ^{1}H NMR (400 MHz, $CDCl_3$) δ 2.20 (s, 3H), 4.56 (brs, 2H), 5.05 (s, 2H), 6.84 (s, 1H), 7.3-7.5 (m, 5H), 7.51 (s, 1H). ^{13}C NMR (100 MHz, $CDCl_3$) δ 17.81, 70.12, 118.35, 122.98, 127.57, 128.22, 128.65, 136.39, 138.27, 141.37, 148.12. Elemental analysis calcd for $C_{13}H_{14}N_2O$: C 72.87%, H 6.59%, N 13.07%;

found: C 72.78%, H 6.60%, N 13.00%. HR-MS: calcd for $C_{13}H_{15}N_2O$ ($[M+H]^+$) 215.1179; found 215.1175.

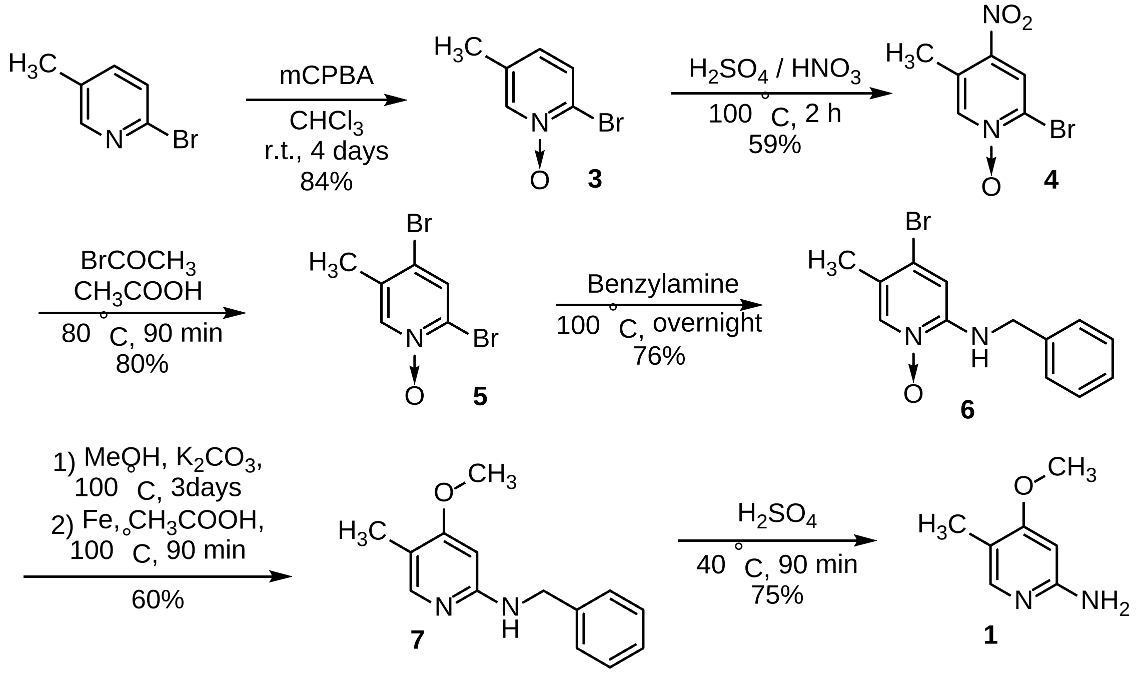

Fig. S2 Synthetic route of 2-amino-4-methoxy-5-methylpyridine (**1**).

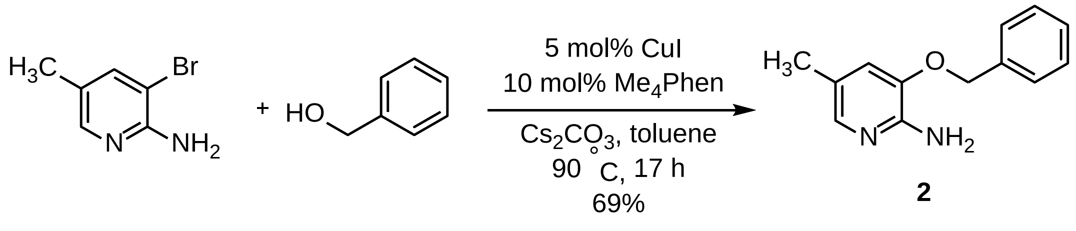

Fig. S3 Synthesis of 2-amino-3-benzoyloxy-5-methylpyridine (**2**)
