# Supplementary material for: Qualitative analysis of 7- and 8-hydroxyzolpidem and discovery of novel zolpidem metabolites in postmortem urine using liquid chromatography–tandem mass spectrometry
Source: Forensic Toxicol. 2022 Jan 4;40(2):263–77. doi: 10.1007/s11419-021-00611-9 (PMC9715527; doi:10.1007/s11419-021-00611-9)
Supplement: Supplementary file 2 — Supplementary file2 (PDF 389 kb) [file 11419_2021_611_MOESM2_ESM.pdf]

Qualitative analysis of 7- and 8-hydroxyzolpidem and discovery of novel zolpidem metabolites in postmortem urine using liquid chromatography-tandem mass spectrometry

Koji Yamaguchi[1] · Hajime Miyaguchi[2] · Youkichi Ohno[1] · Yoshimasa Kanawaku[1]

Supplementary Information: Table S1, S2 and Figure S1.

Table S1. *m/z* values of ions observed at the LC-QqTOFMS analysis, and their estimated formulas.

Peak	Scan mode	Precursor *m/z*	Observed *m/z*	Estimated formula	Calculated *m/z*	Error (mDa)
A	Scan	–	342.1813	$C_{19}H_{24}N_3O_3$	342.1812	0.06
	Product ion scan	342.1812	324.1705	$C_{19}H_{22}N_3O_2$	324.1707	0.19
			279.1134	$C_{17}H_{15}N_2O_2$	279.1128	0.55
			251.1180	$C_{16}H_{15}N_2O$	251.1179	0.11
B	Scan	–	427.1800	$C_{22}H_{27}N_4O_3S$	427.1798	0.16
	Product ion scan	427.1798	340.1479	$C_{19}H_{22}N_3OS$	340.1478	0.07
			295.0900	$C_{17}H_{15}N_2OS$	295.0900	0.05
			267.0952	$C_{16}H_{15}N_2S$	267.0951	0.15
C	Scan	–	445.1906	$C_{22}H_{29}N_4O_4S$	445.1904	0.18
	Product ion scan	445.1904	427.1800	$C_{22}H_{27}N_4O_3S$	427.1798	0.15
			340.1479	$C_{19}H_{22}N_3OS$	340.1478	0.11
			324.1707	$C_{19}H_{22}N_3O_2$	324.1707	0.09
			308.1758	$C_{19}H_{22}N_3O$	308.1757	0.04
			279.1140	$C_{17}H_{15}N_2O_2$	279.1128	1.21
			251.1181	$C_{16}H_{15}N_2O$	251.1179	0.16
			235.1232	$C_{16}H_{15}N_2$	235.1230	0.22
D3	Scan	–	500.2029	$C_{25}H_{30}N_3O_8$	500.2027	0.16
	Product ion scan	500.2027	324.1709	$C_{19}H_{22}N_3O_2$	324.1707	0.22
			279.1137	$C_{17}H_{15}N_2O_2$	279.1128	0.85
			252.1259	$C_{16}H_{16}N_2O$	252.1257	0.23
			251.1181	$C_{16}H_{15}N_2O$	251.1179	0.17
E3	Scan	–	518.2135	$C_{25}H_{32}N_3O_9$	518.2133	0.18
	Product ion scan	518.2133	342.1812	$C_{19}H_{24}N_3O_3$	342.1812	0.02
			324.1707	$C_{19}H_{22}N_3O_2$	324.1707	0.00
			296.1757	$C_{18}H_{22}N_3O$	296.1757	−0.03
			279.1132	$C_{17}H_{15}N_2O_2$	279.1128	0.36
			251.1180	$C_{16}H_{15}N_2O$	251.1179	0.12

LC-QqTOFMS: liquid chromatography-quadrupole time-of-flight mass spectrometry.

Table S2. Changes in peak areas upon glucuronidase treatment.

Compound	Peak	Peak area		Change* (%)
		Glu −	Glu +	
ZOL		13,117,533	12,564,540	96
M1		218,418,183	215,358,442	99
M2		24,997,092	24,535,628	98
M3		225,956	317,248	140
M4		11,751,676	20,744,834	177
7OHZ		15,879,802	31,442,856	198
8OHZ		345,775	3,050,829	880
ZDHD	A	9,754,640	74,361,618	762
Z-Cys	B	5,775,410	5,821,221	101
Z-H_2OCys	C	6,946,591	6,098,903	88
Z-OGlus	D1	837,710	n.d.	0
	D2	300,609	n.d.	0
	D3	39,502,000	62,245	0.16
	D4	6,109,127	8,512	0.14
	D5	9,809,327	14,042	0.14
ZDHD-Glus	E1	3,697,998	n.d.	0
	E2	365,611	763	0.21
	E3	35,066,186	84,082	0.24

Peak areas in Glu + and Glu – columns represent averages of peak areas (*n* =3) with and without the glucuronidase treatment.

*: Values are calculated by dividing the peak areas with glucuronidase treatment by those without the treatment.

n.d.: not detected.

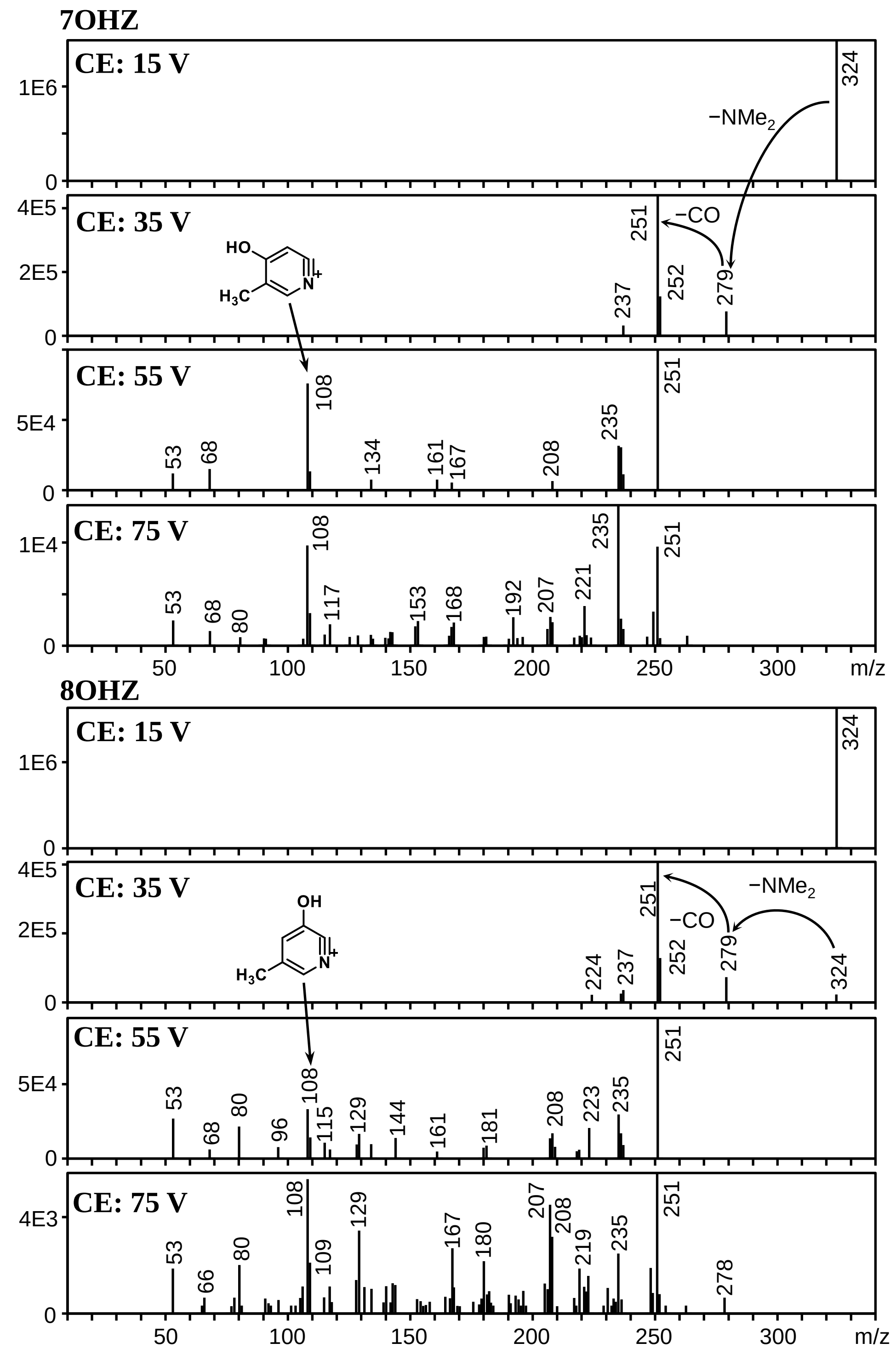

Fig. S1 Mass spectra of 7OHZ and 8OHZ at various collision energies (CEs)
