# Supplementary material for: Qualitative analysis of 7- and 8-hydroxyzolpidem and discovery of novel zolpidem metabolites in postmortem urine using liquid chromatography–tandem mass spectrometry
Source: Forensic Toxicol. 2022 Jan 4;40(2):263–77. doi: 10.1007/s11419-021-00611-9 (PMC9715527; doi:10.1007/s11419-021-00611-9)
Supplement: Supplementary file 3 — Supplementary file3 (PDF 7104 kb) [file 11419_2021_611_MOESM3_ESM.pdf]

Supplementary Information

Qualitative analysis of 7- and 8- hydroxyzolpidem and discovery of novel zolpidem metabolites in postmortem urine using liquid chromatography-tandem mass spectrometry

Koji Yamaguchi · Hajime Miyaguchi · Youkichi Ohno · Yoshimasa Kanawaku

NMR data of compound **1** to **7**, 7OMeZ, 8OBzZ, 7OHZ hydrobromide, and 8OHZ tosylate.

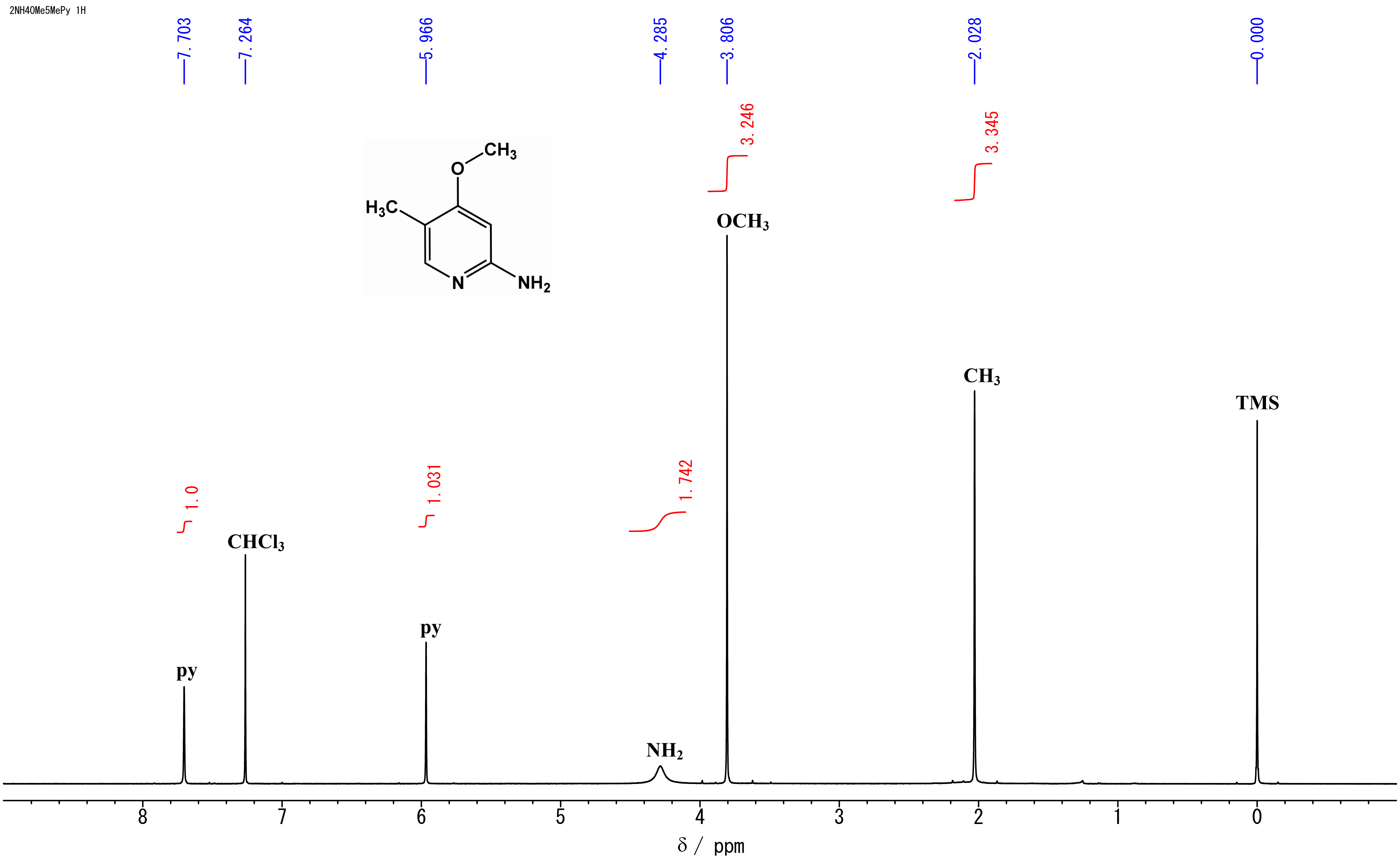

Fig. 1. ^{1}H NMR spectrum of 2-amino-4-methoxy-5-methylpyridine (**1**, 400 MHz, $CDCl_3$).

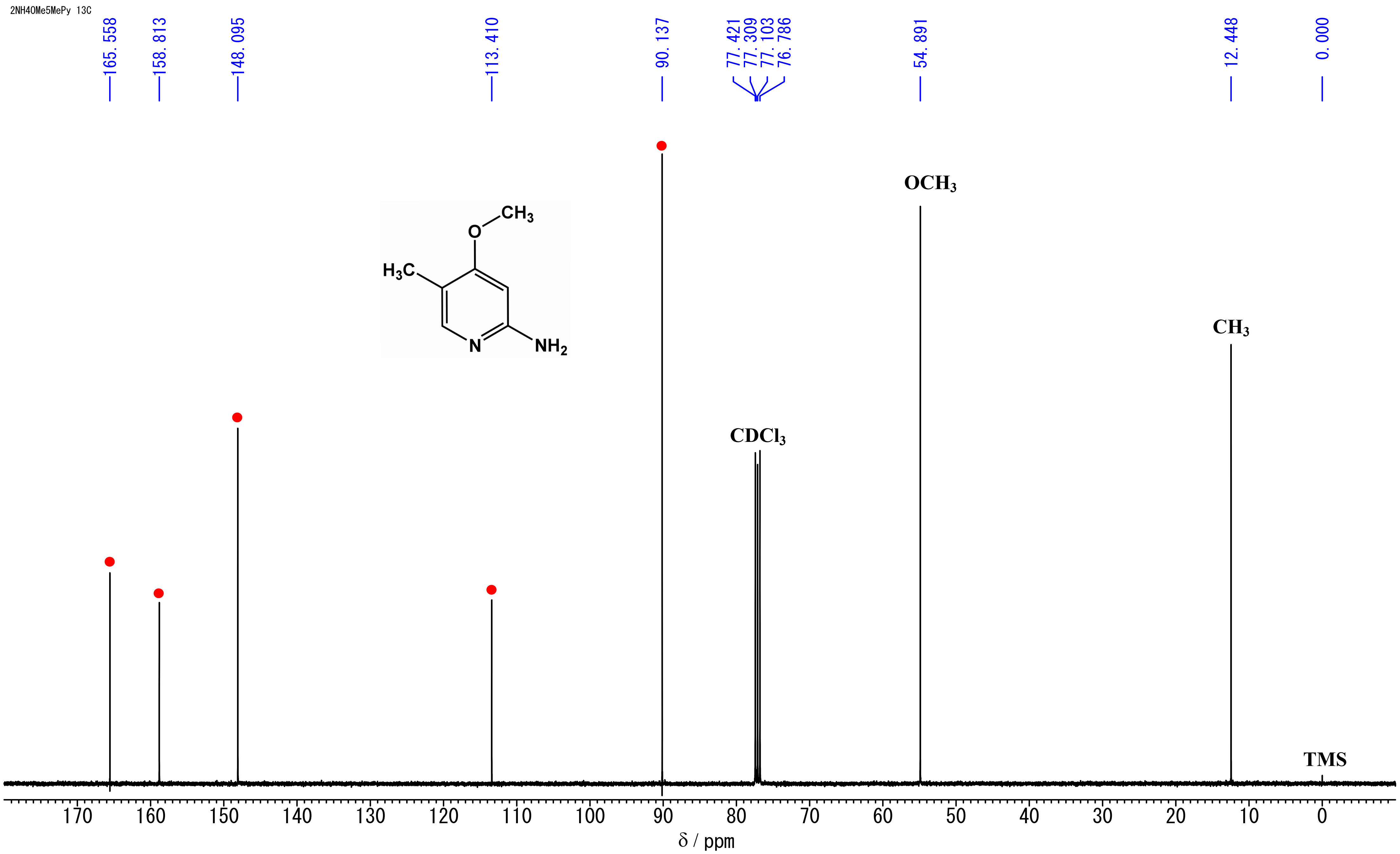

Fig. 2. ^{13}C NMR spectrum of **1** (100 MHz, $CDCl_3$).

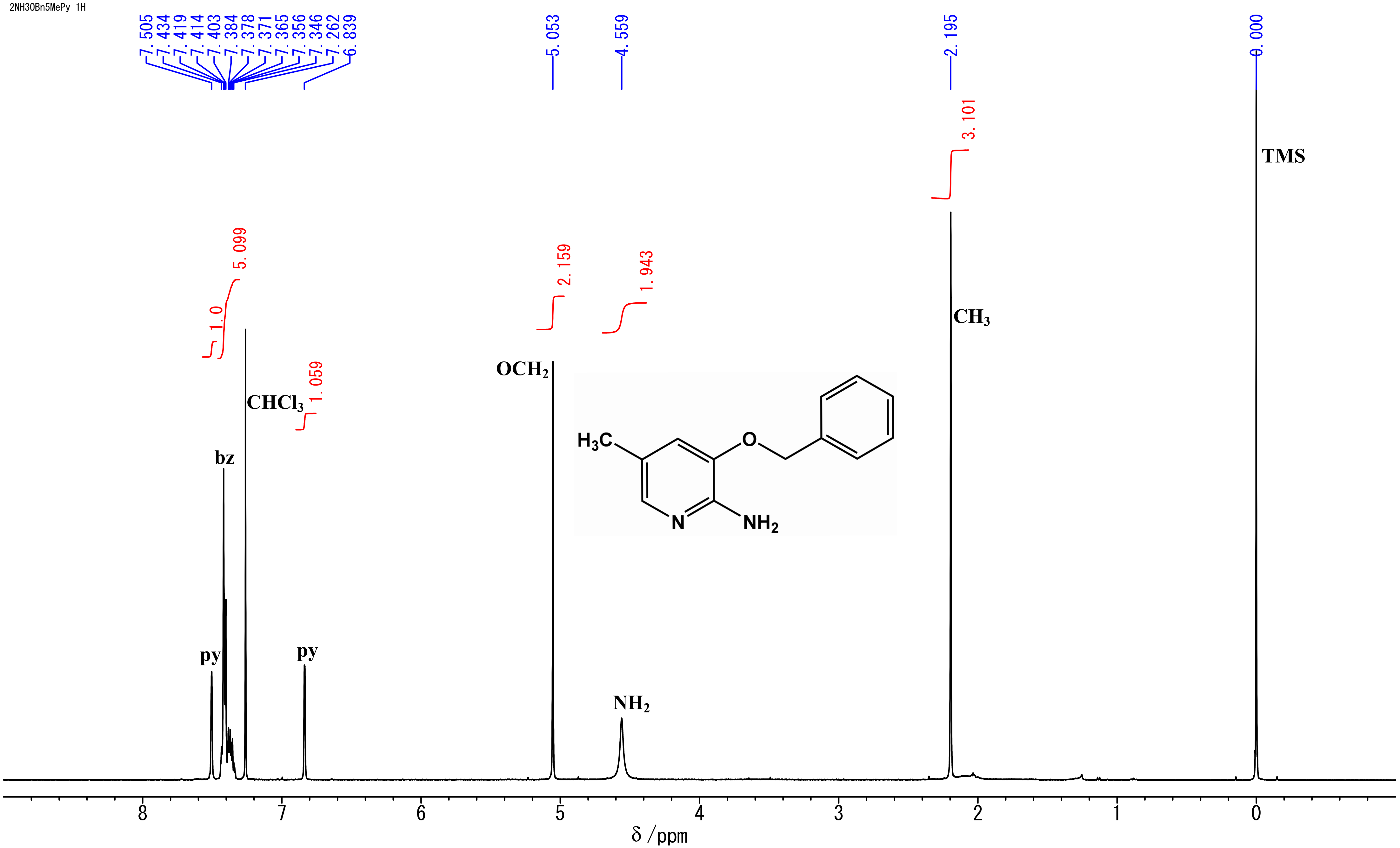

Fig. 3. ^{1}H NMR spectrum of 2-amino-4-methoxy-5-methylpyridine (**2**, 400 MHz, $CDCl_3$).

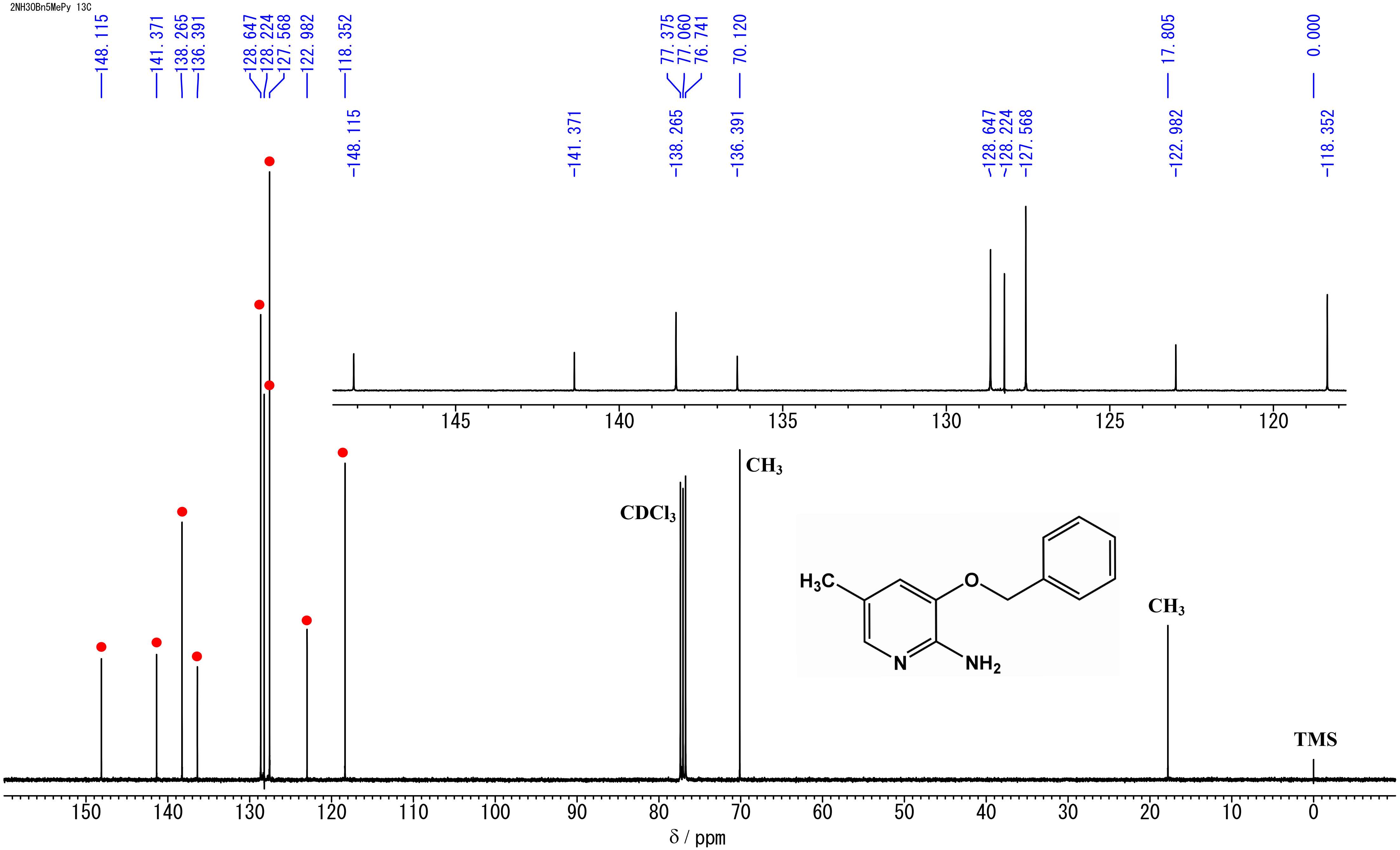

Fig. 4. ^{13}C NMR spectrum of **2** (100 MHz, $CDCl_3$).

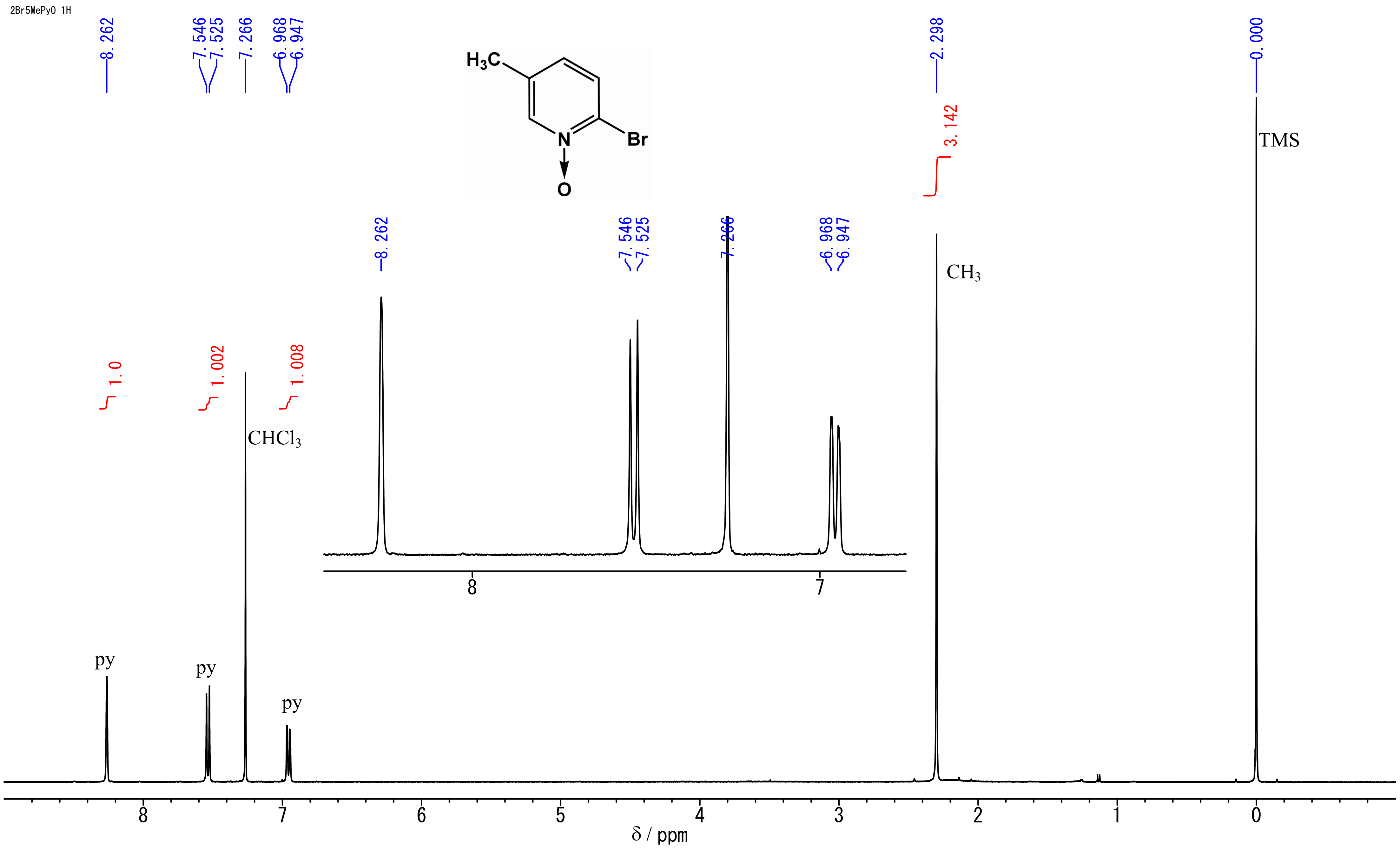

Fig. 5. ^{1}H NMR spectrum of 2-bromo-5-methylpyridine oxide (**3**, 400 MHz, $CDCl_3$).

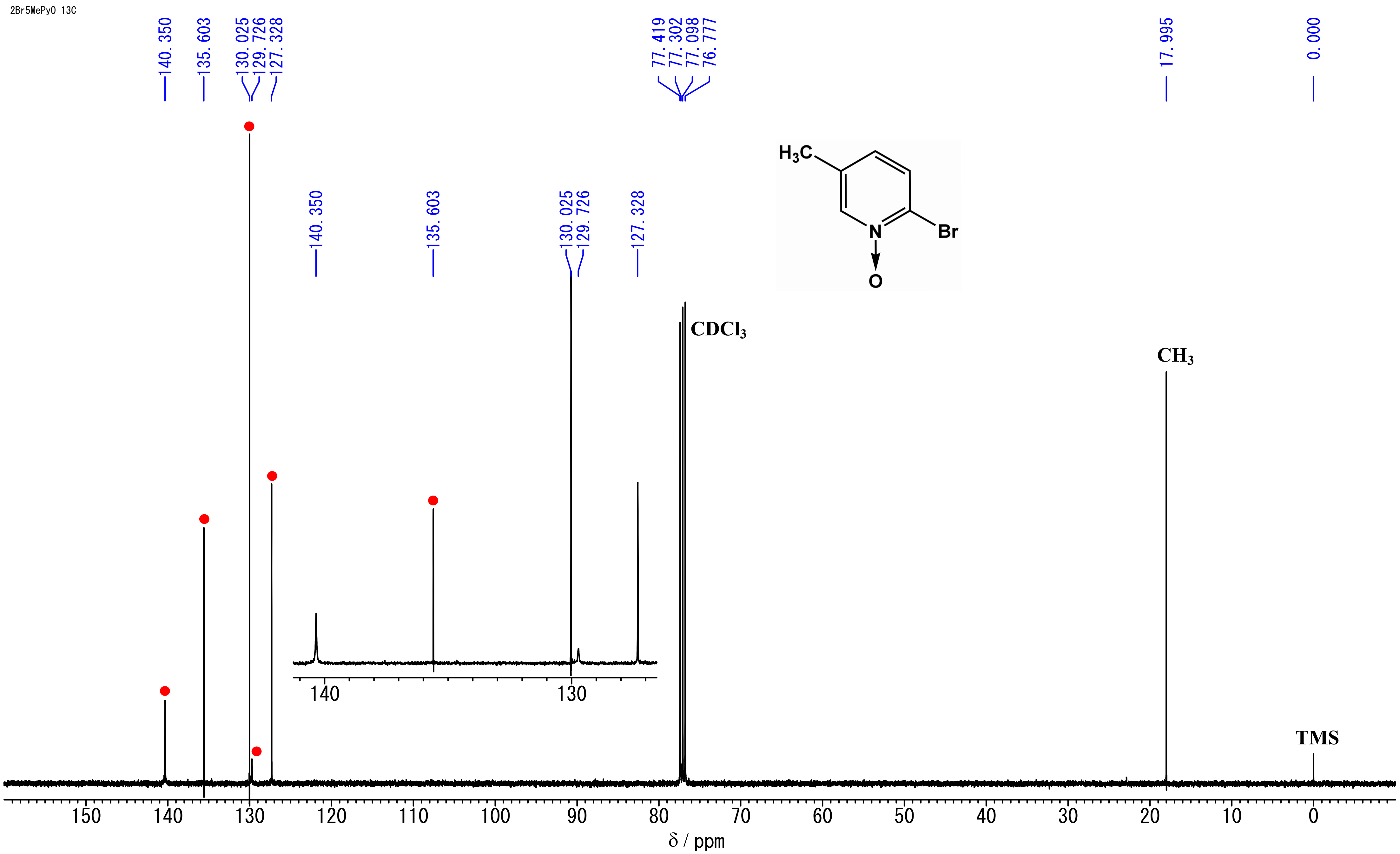

Fig. 6. ^{13}C NMR spectrum of **3** (100 MHz, $CDCl_3$).

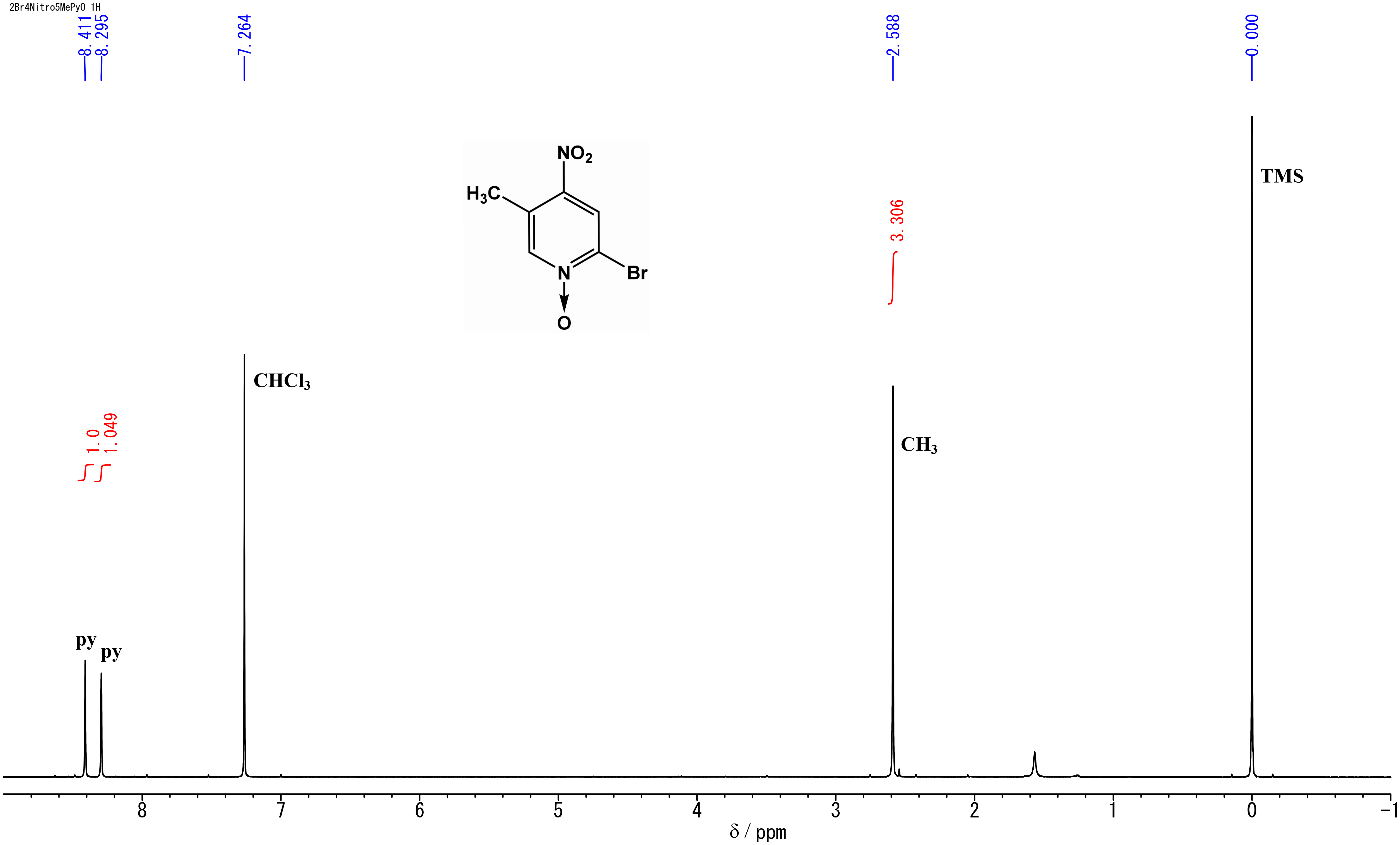

Fig. 7. ^{1}H NMR spectrum of 2-bromo-4-nitro-5-methylpyridine oxide (**4**, 400 MHz, $CDCl_3$).

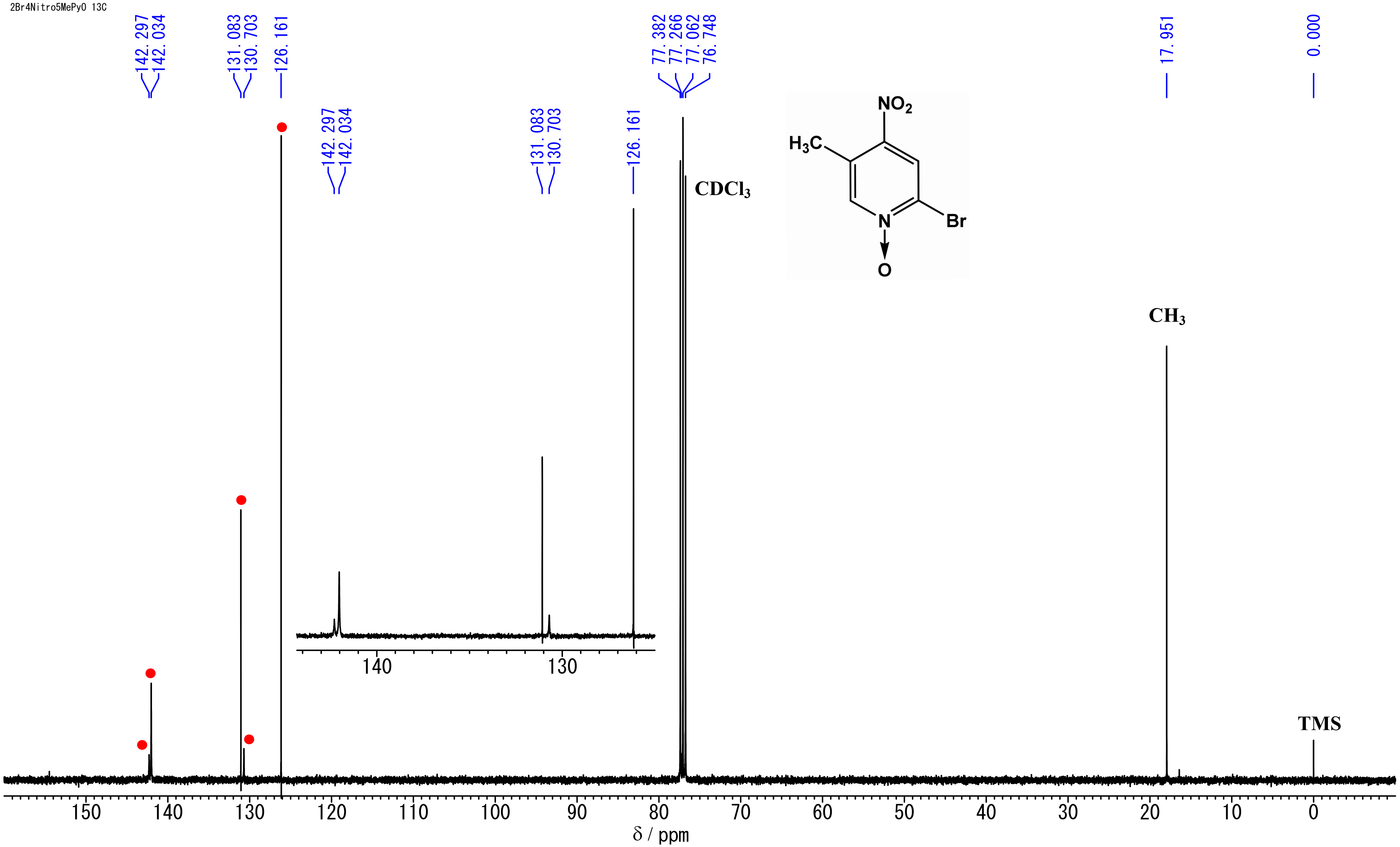

Fig. 8. ^{13}C NMR spectrum of **4** (100 MHz, $CDCl_3$).

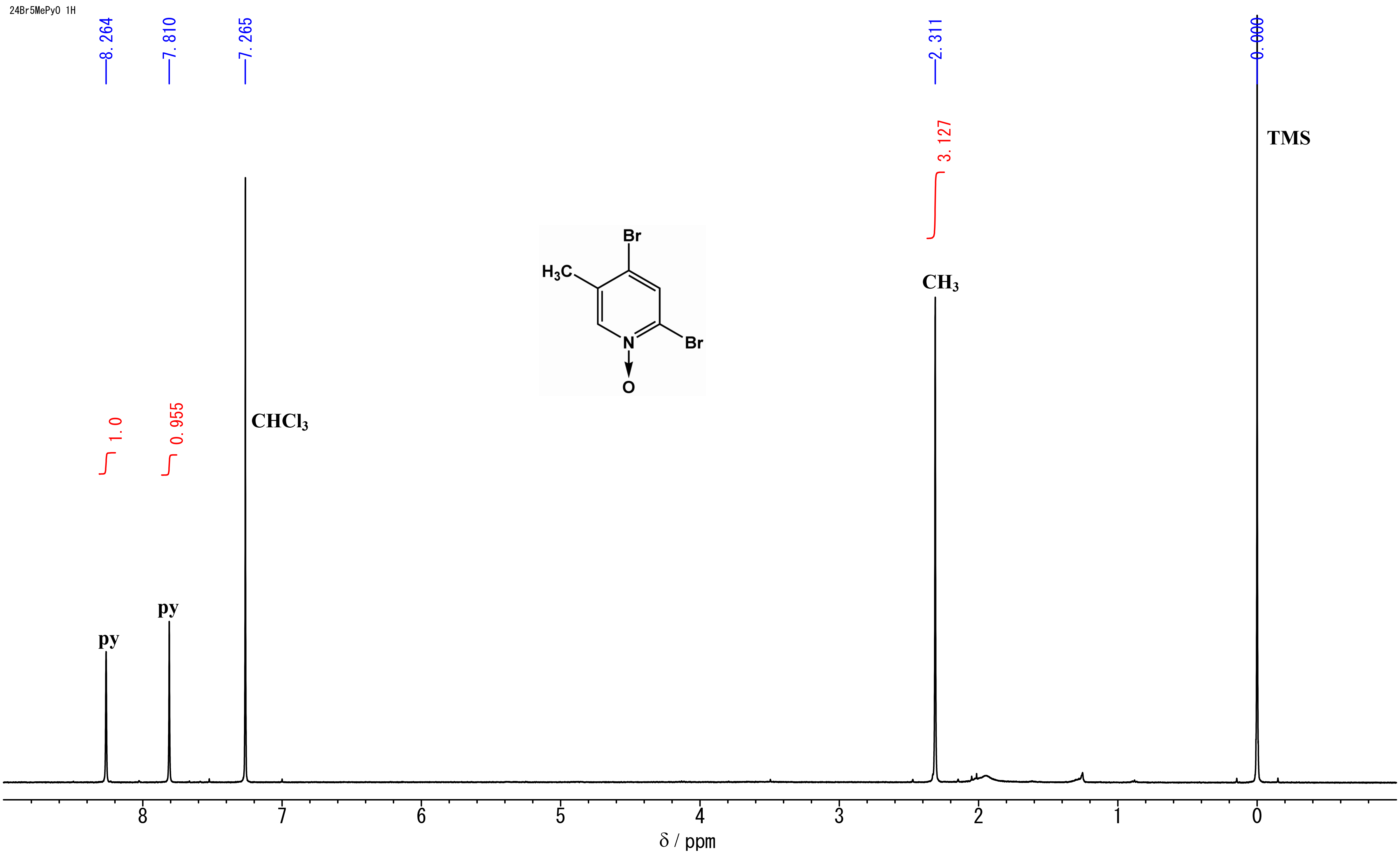

Fig. 9. ^{1}H NMR spectrum of 2,4-dibromo-5-methylpyridine oxide (**5**, 400 MHz, $CDCl_3$).

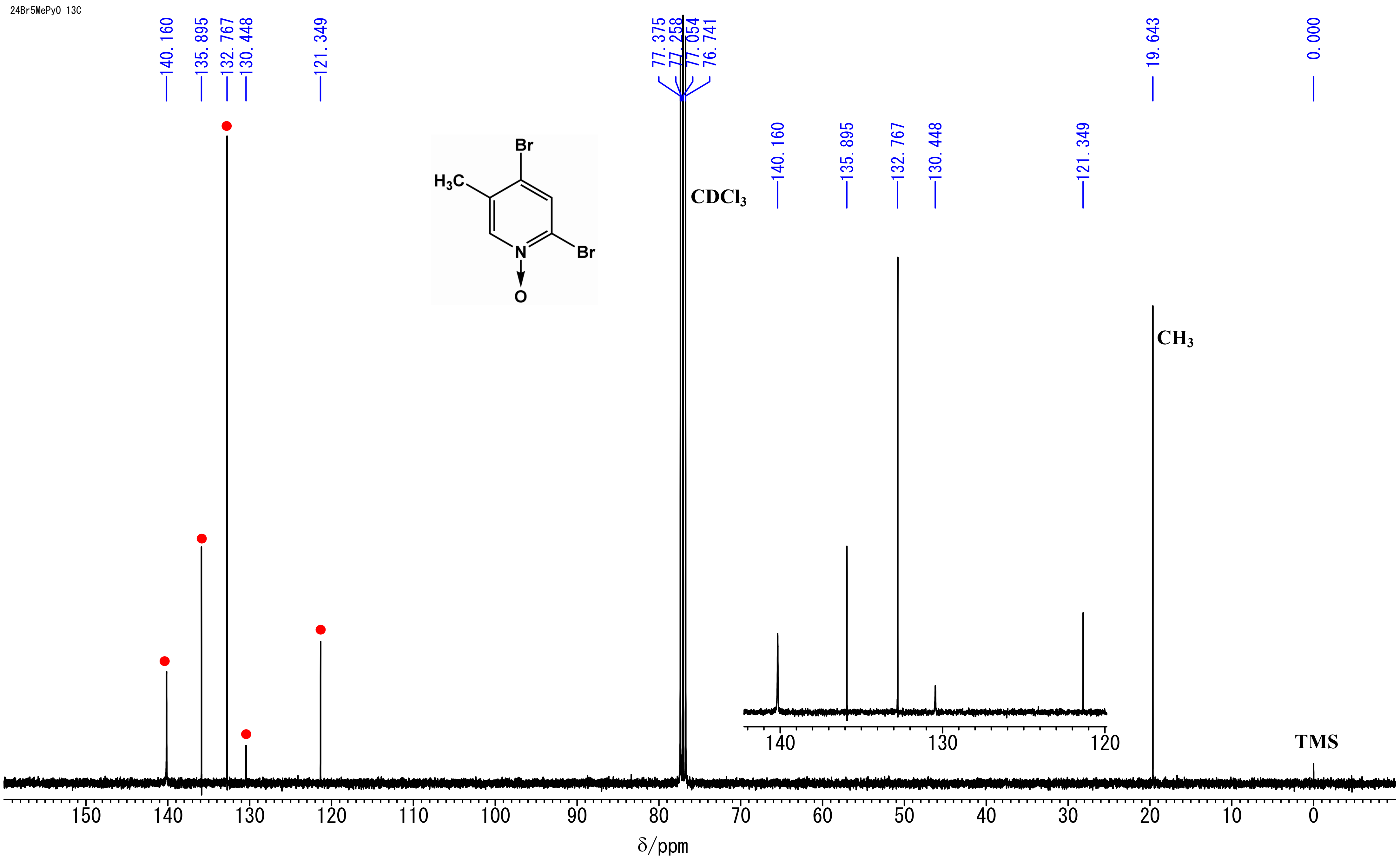

Fig. 10. ^{13}C NMR spectrum of **5** (100 MHz, $CDCl_3$).

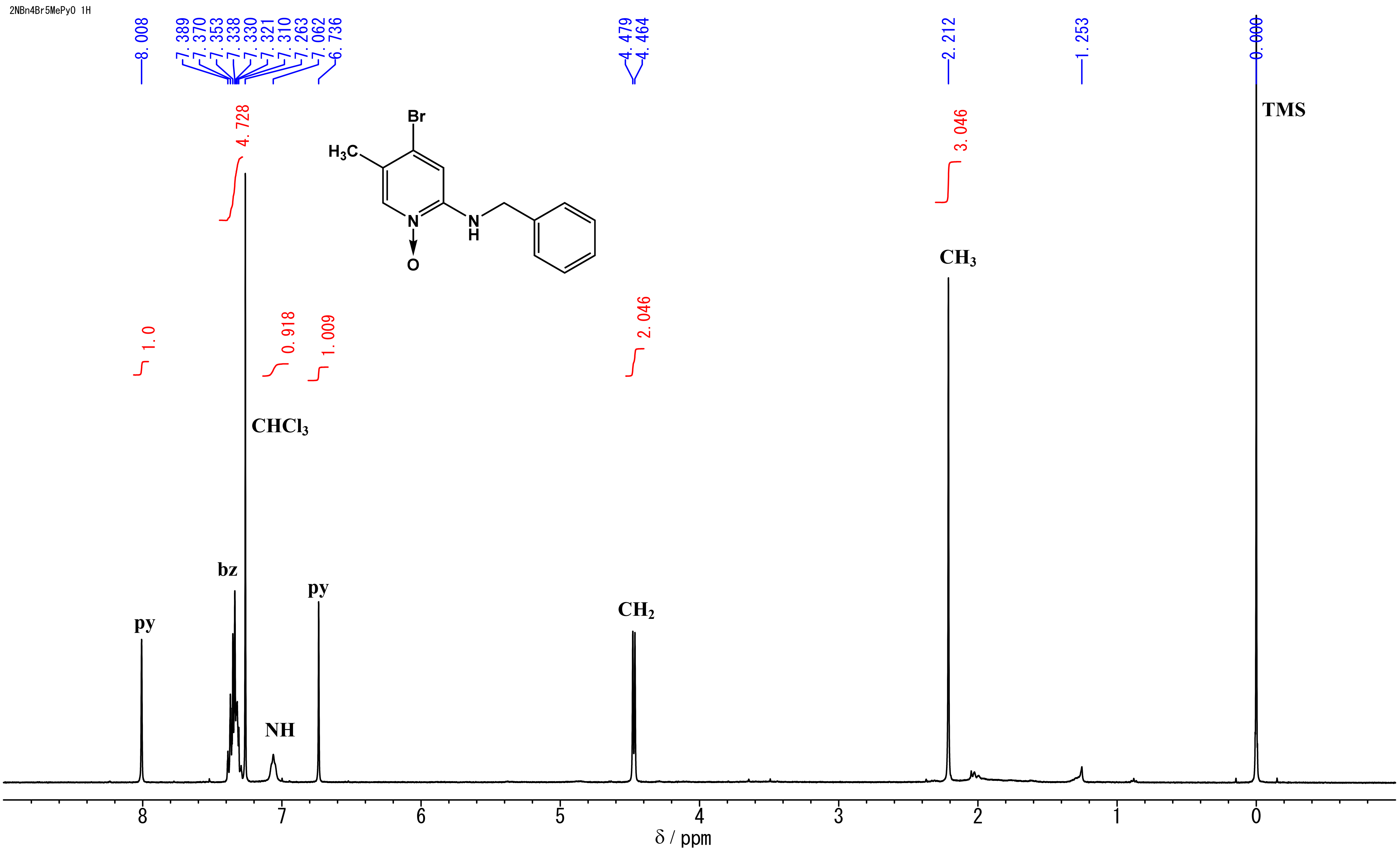

Fig. 11. ^{1}H NMR spectrum of 2-benzylamino-4-bromo-5-methylpyridine oxide (**6**, 400 MHz, $CDCl_3$).

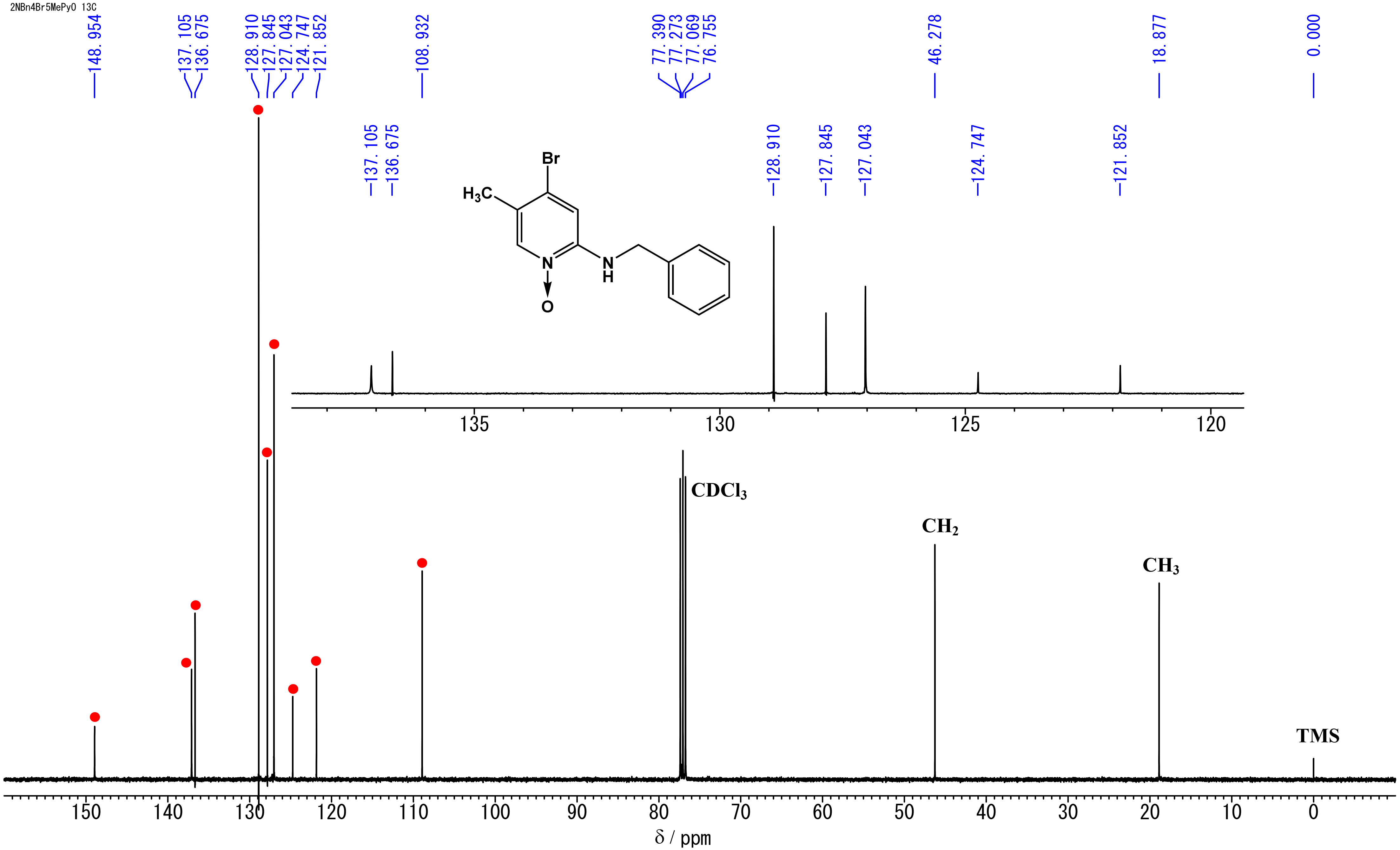

Fig. 12. ^{13}C NMR spectrum of **6** (100 MHz, $CDCl_3$).

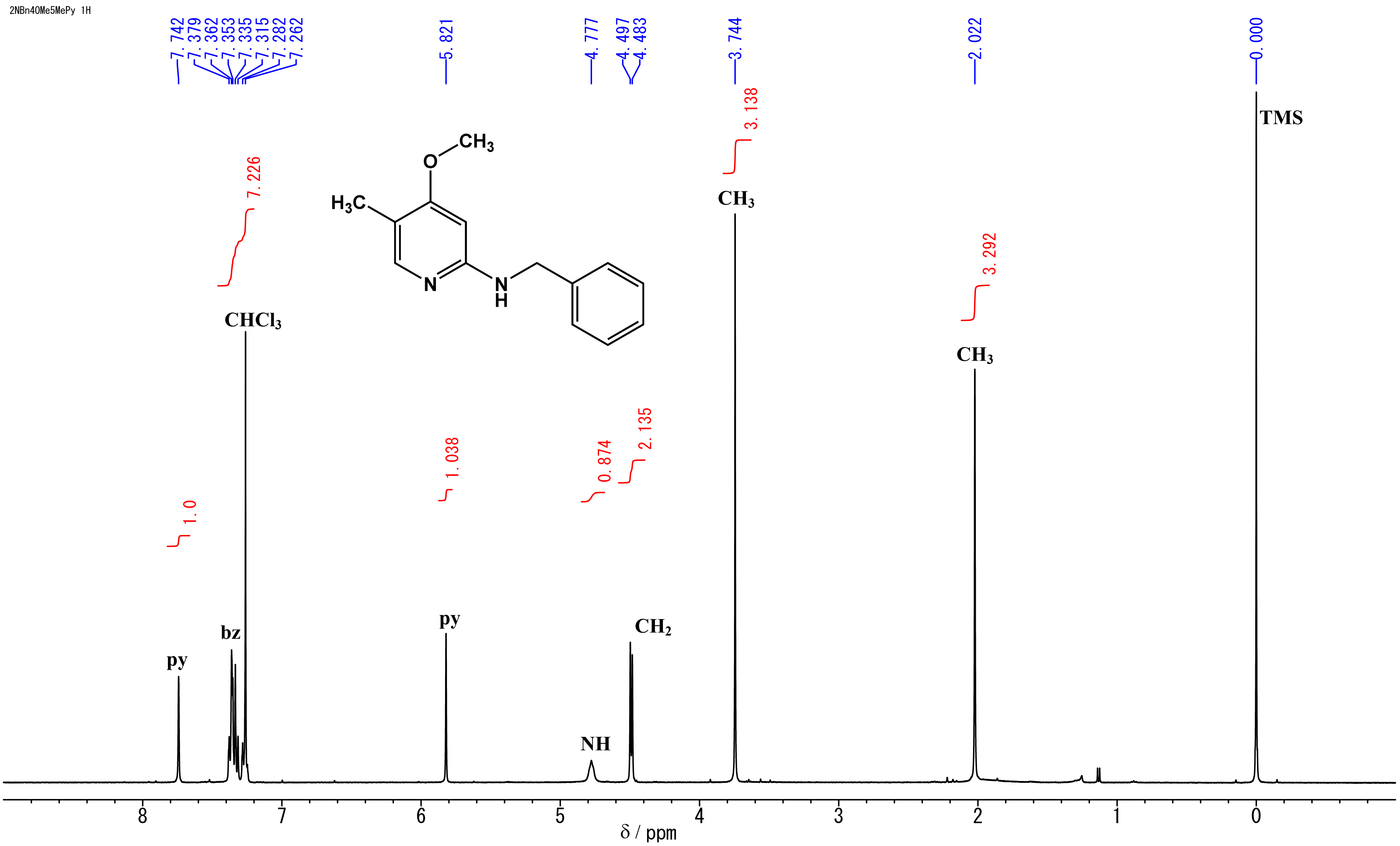

Fig. 13. ^{1}H NMR spectrum of 2-benzylamino-4-methoxy-5-methylpyridine (**7**, 400 MHz, $CDCl_3$).

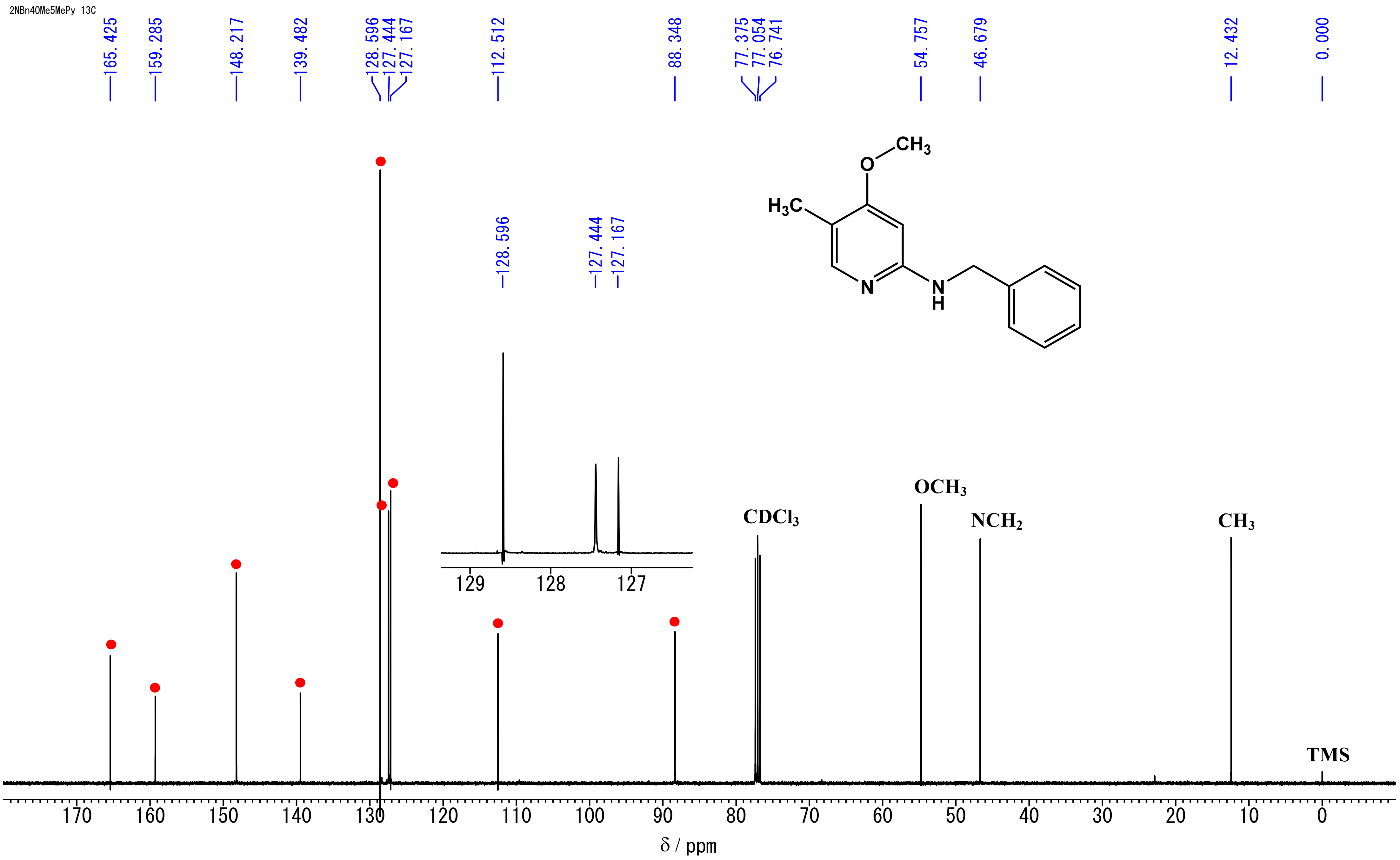

Fig. 14. ^{13}C NMR spectrum of **7** (100 MHz, $CDCl_3$).

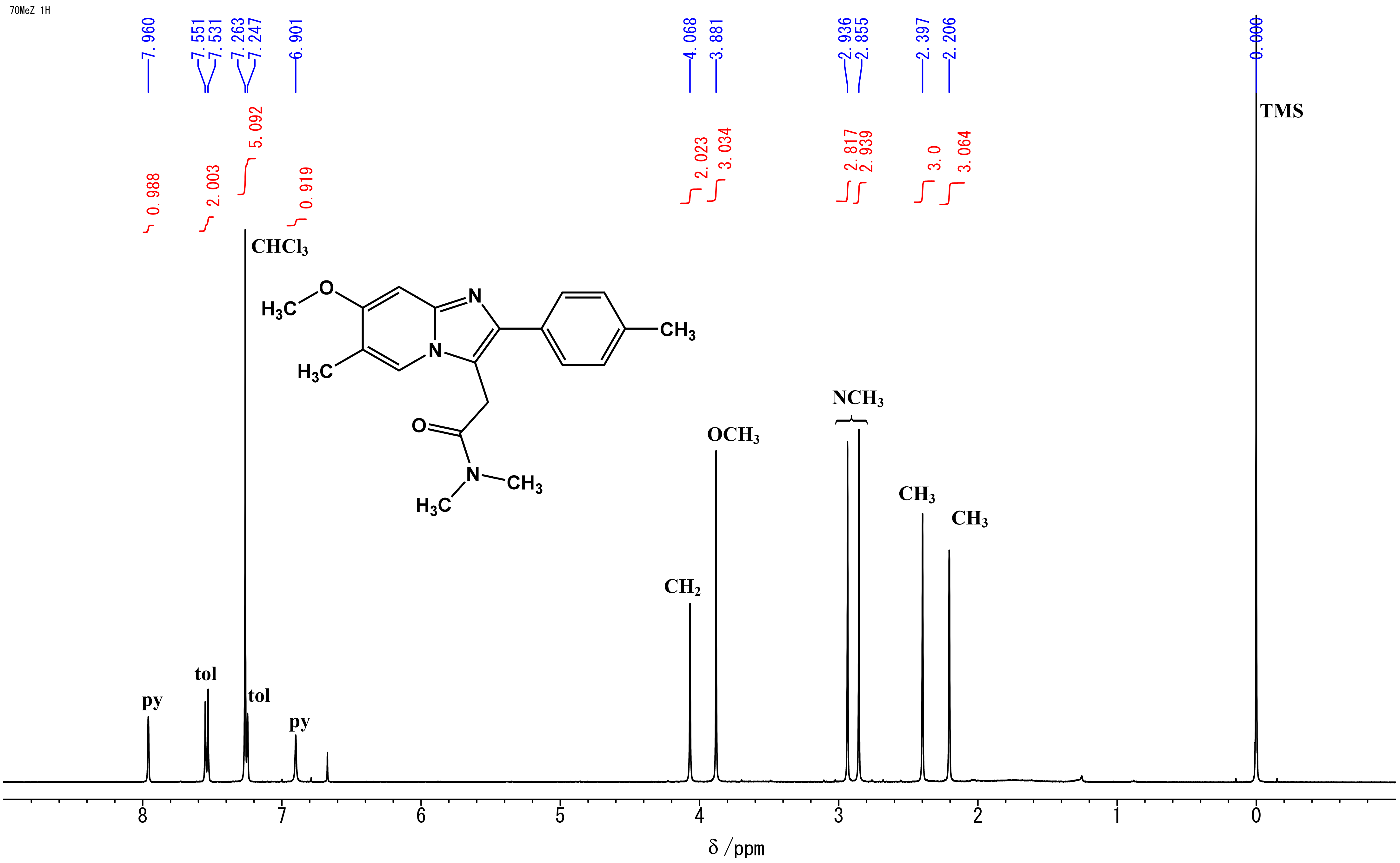

Fig. 15. ^{1}H NMR spectrum of 7OMeZ (400 MHz, $CDCl_3$).

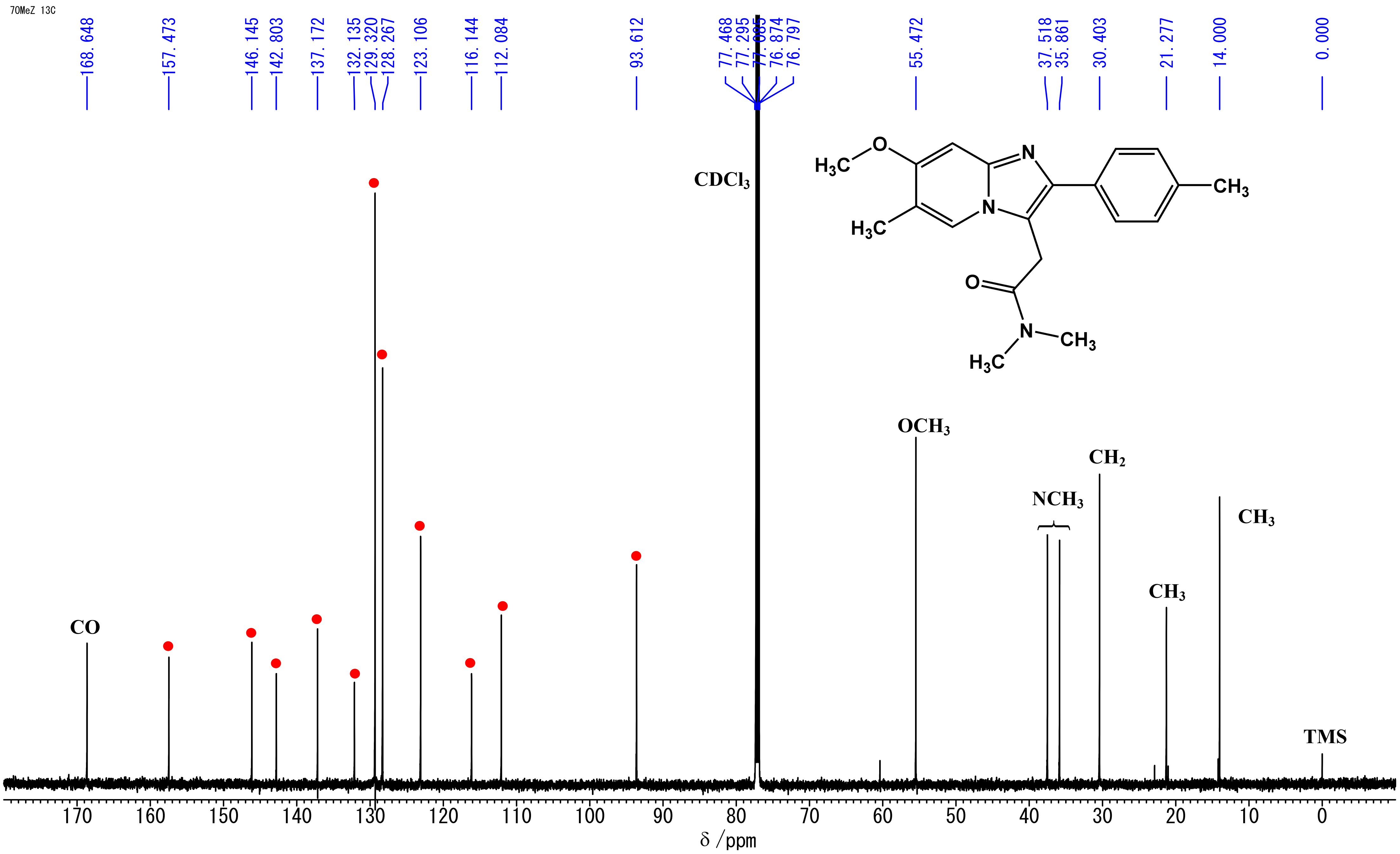

Fig. 16. ^{13}C NMR spectrum of 7OMeZ (151 MHz, $CDCl_3$).

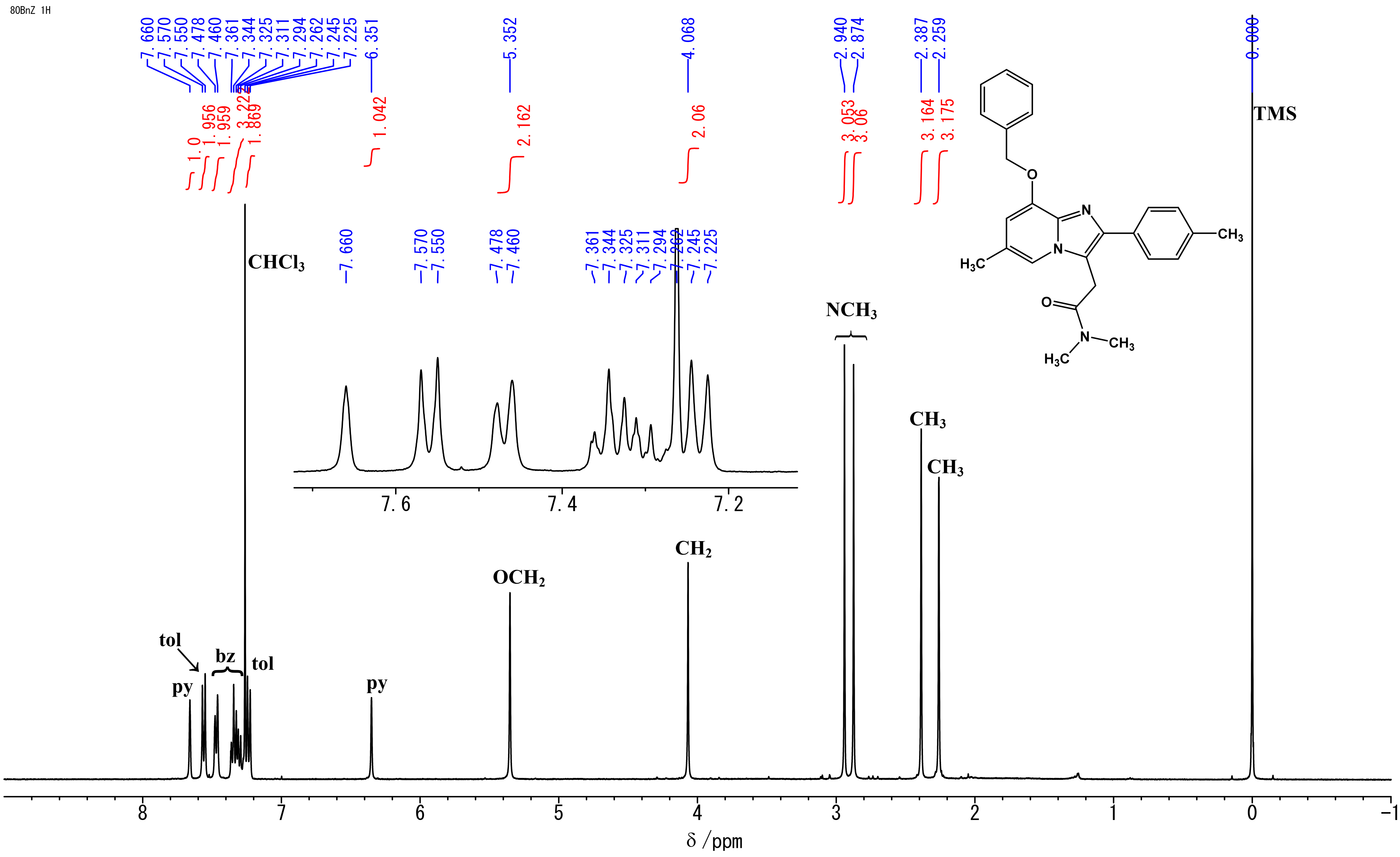

Fig. 17. ^{1}H NMR spectrum of 8OBzZ (400 MHz, $CDCl_3$).

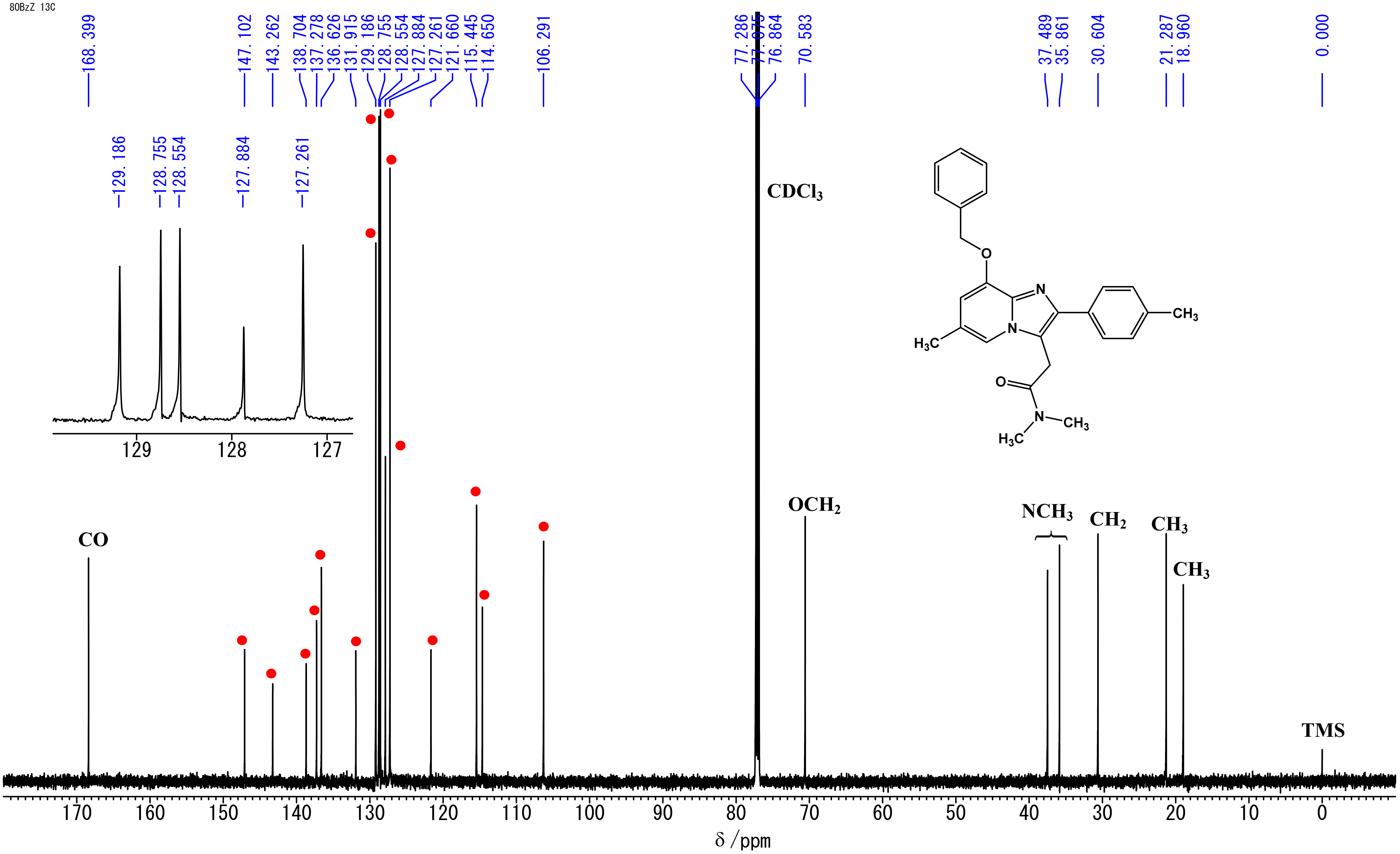

Fig. 18. ^{13}C NMR spectrum of 8OBzZ (151 MHz, $CDCl_3$).

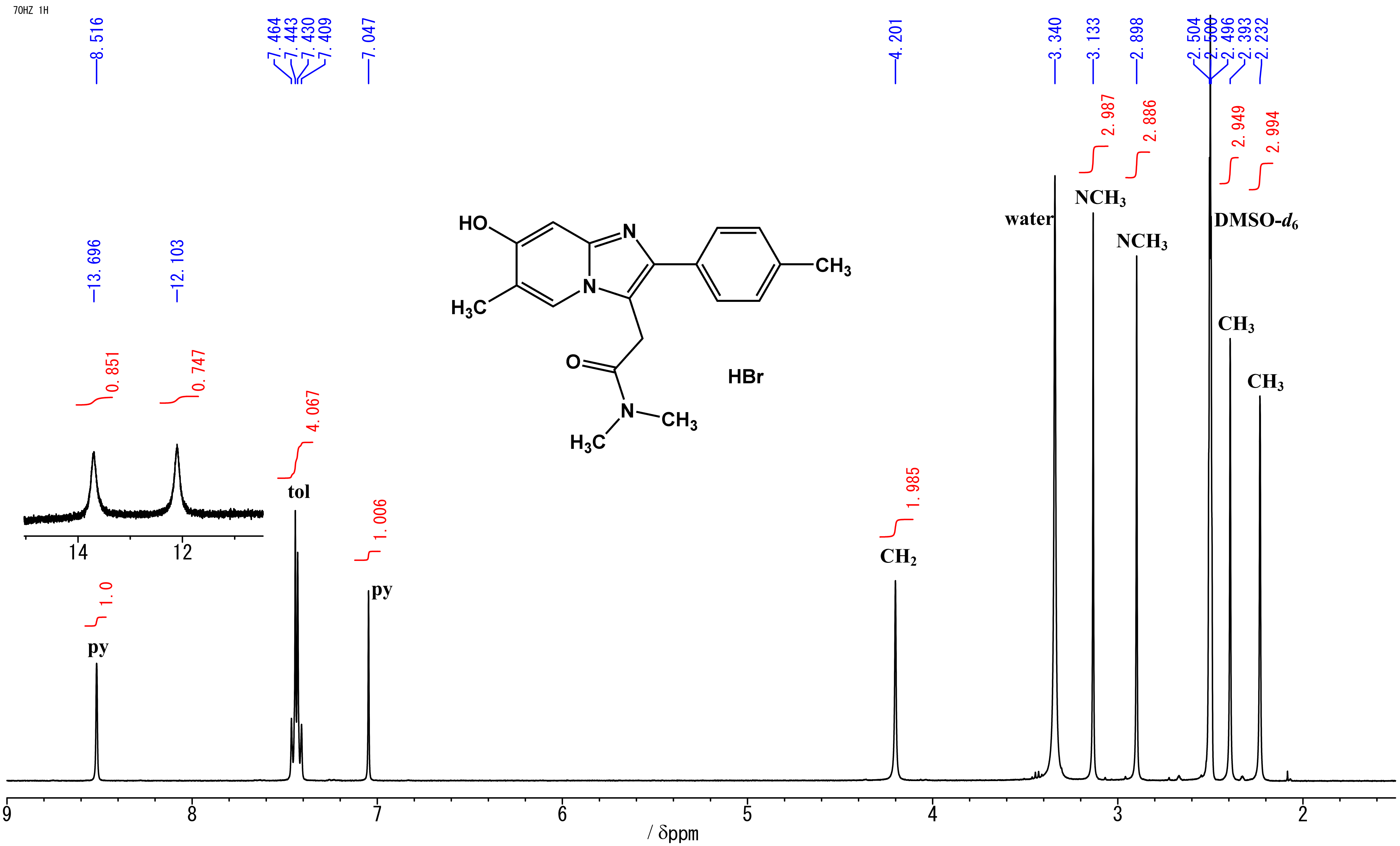

Fig. 19. ^{1}H NMR spectrum of 7OHZ hydrobromide (400 MHz, DMSO-d_6).

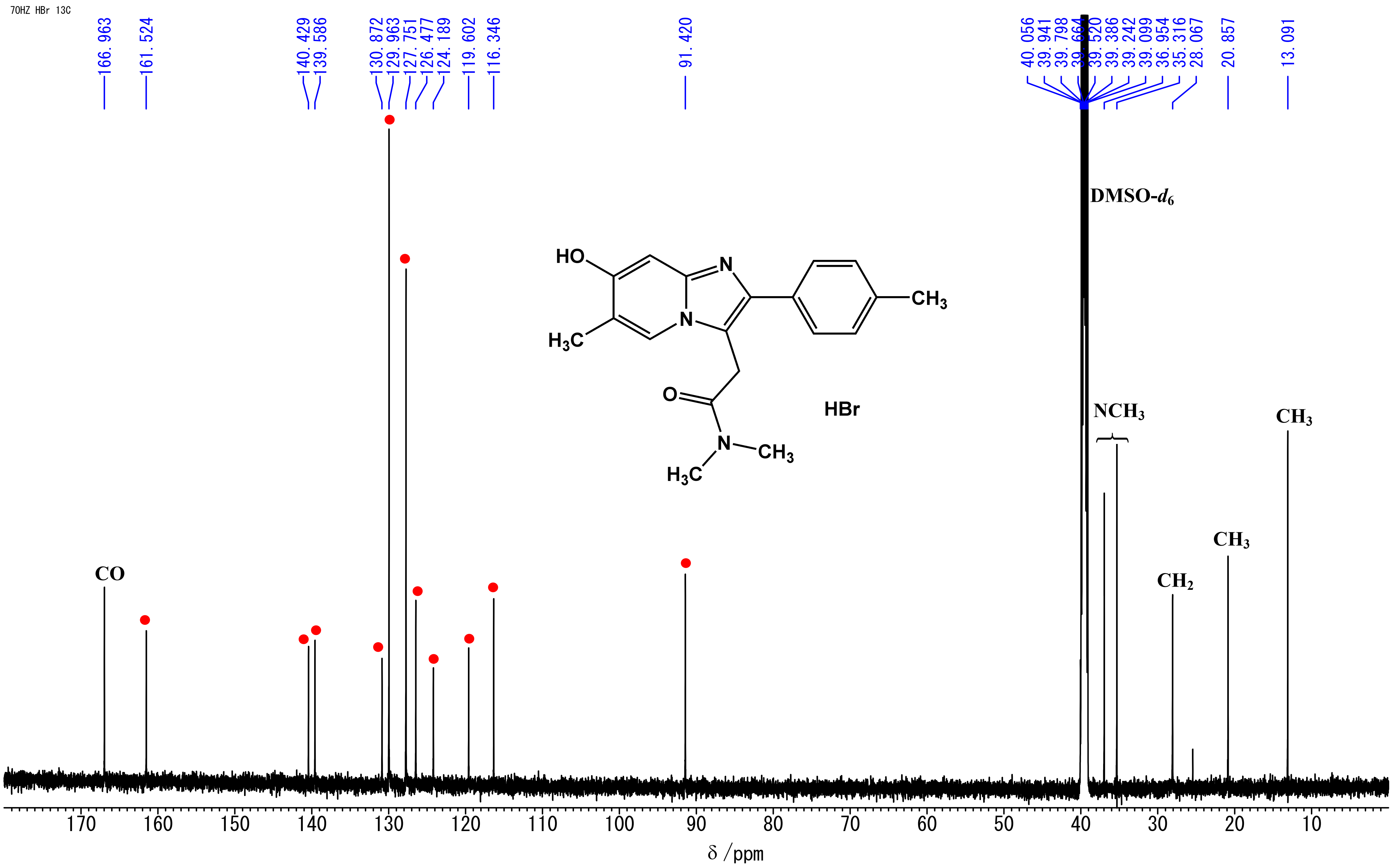

Fig. 20. ^{13}C NMR spectrum of 7OHZ hydrobromide (151 MHz, DMSO-d_6).

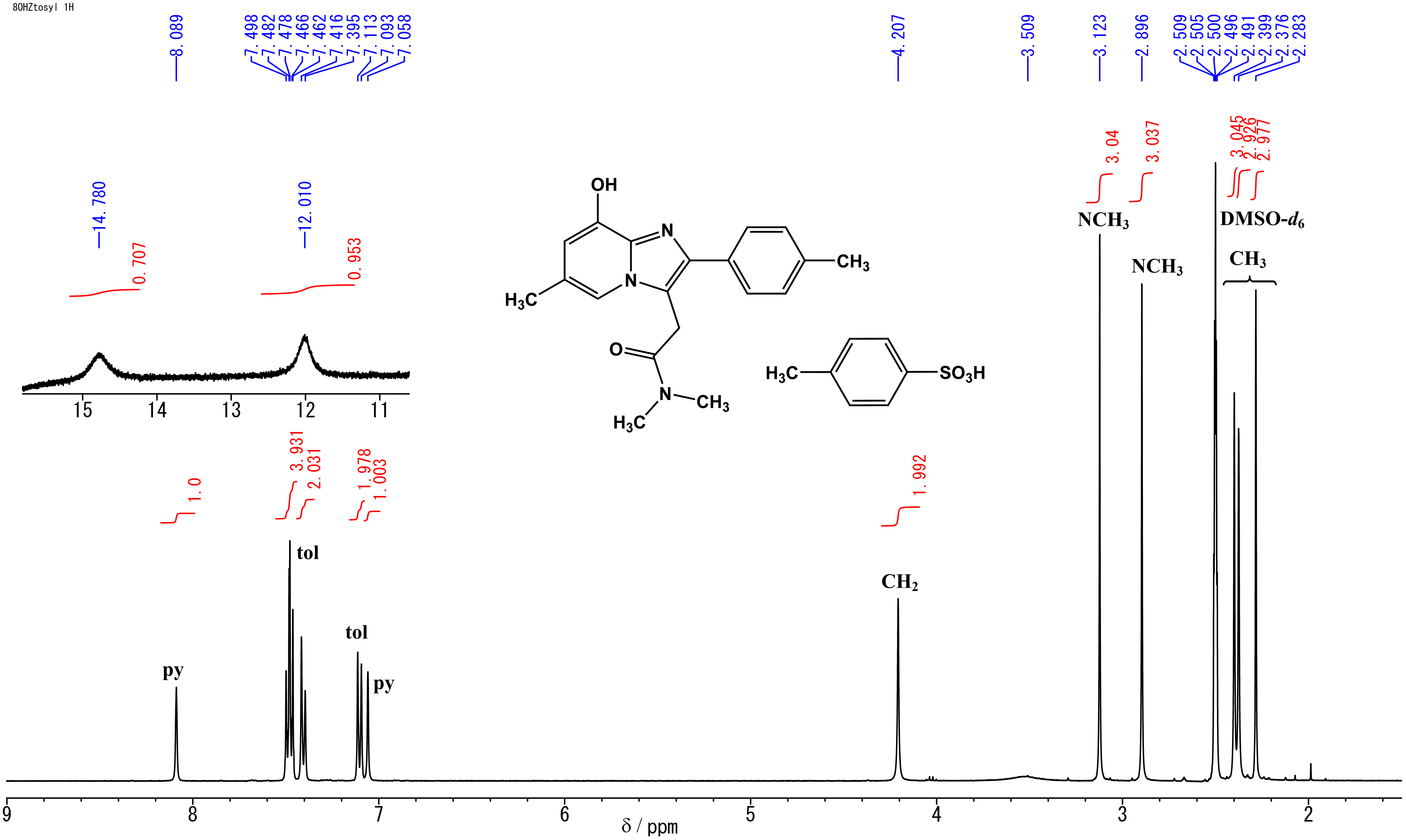

Fig. 21. ^{1}H NMR spectrum of 8OHZ tosylate (400 MHz, DMSO-d_6).

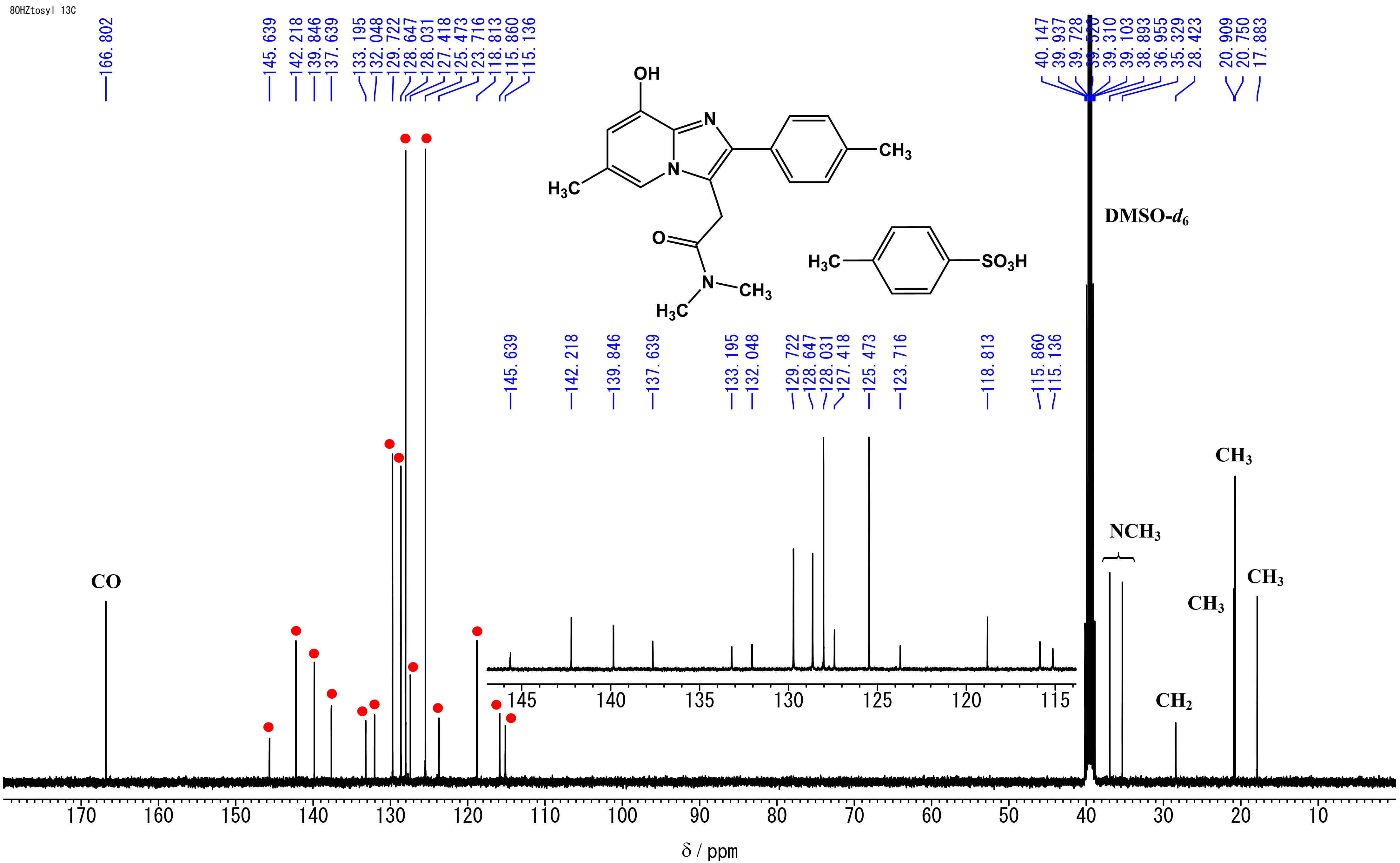

Fig. 22. ^{13}C NMR spectrum of 8OHZ tosylate (100 MHz, DMSO-d_6).
